# Emergent technologies in clinical neurophysiology to study the neuromuscular system: IFCN handbook chapter

**DOI:** 10.1016/j.cnp.2026.09.003

**Published:** 2026-09-18

**Authors:** H. Tankisi, H. Bostock, L.D. Hobson-Webb, D. Holder, J. Howells, H. Olausson, S.B. Rutkove, N. Shahrizaila, W. Z'Graggen, J. Valls-Sole, J. Cole

**Affiliations:** a Department of Clinical Neurophysiology and Medicine, Aarhus University Hospital, Aarhus, Denmark; b Department of Neuromuscular Diseases, UCL Queen Square Institute of Neurology, Queen Square, WC1N 3BG, London, United Kingdom; c Department of Neurology/Neuromuscular Division, Duke University Health System, Durham, NC, United States; d Department of Medical Physics and Biomedical Engineering, University College London, London, United Kingdom; e Central Clinical School, Faculty of Medicine and Health, University of Sydney, Sydney, Australia; f Department of Biomedical and Clinical Sciences, Linköping University, Linköping, Sweden; g Department of Neurology, Beth Israel Deaconess Medical Center, Harvard Medical School, Boston, MA, USA; h Division of Neurology, Department of Medicine, University of Malaya, Kuala Lumpur 50603, Malaysia; i Departments Neurology and Neurosurgery, Inselspital, Bern University Hospital, University of Bern, Bern, Switzerland; j IDIBAPS (Biomedical Research Institute August Pi i Sunyer), Barcelona, Spain; k Department of Clinical Neurophysiology, University Hospitals Dorset (Poole), UK; l University of Bournemouth, Poole, UK

**Keywords:** Electromyography, Electrical impedance tomography, Electrical impedance myography, Motor unit number estimation, Axonal excitability testing, Muscle excitability testing, Microneurography, Neuromuscular ultrasound

## Abstract

The present chapter of the new third series IFCN Handbook focuses on how peripheral neurophysiology may develop in the next five years or more. While some are clinical relevant, others are more experimental. The first three sections detail the possible futures of EMG, motor unit number estimation, surface EMG and movement. In a section on magnetoneurography, electrically induced signals are visualized in sensory nerves whilst that on magnetomyography shows how motor units may be identified in greater detail than with surface EMG. Electrical impedance methods for assessing nerve and muscle are considered, with the latter having been used clinically in several neurological conditions already. The section on microneurography describes how single nerve cells' response properties are related to RNA activity from dorsal root ganglion cells, leading to the identification of sixteen peripheral sensory nerve populations. Hyperexcitability syndromes can be difficult to diagnose, so nerve and muscle excitability testing, measuring membrane properties and ion channelopathies, shows clinical promise. The last section details ultrasound methods used in peripheral nerve and muscle disease and shows how ultrasound is becoming a necessary adjunct to nerve conduction studies and EMG. Common considerations between these methods' future include the greater use of artificial intelligence and that being non-invasive means they may be used for both diagnosis and long-term monitoring. Common barriers to wider availability include access to complex software and appropriate machines.

## Highlights

• The ten sections in this review cover updated diagnostic tools and novel techniques.
• This review shows the rich variety of methods used to study peripheral nervous system.
• Common considerations for the future of the methods include greater use of AI.

## 1 Introduction

Not infrequently patients jokingly ask us during nerve conduction studies (NCS) and EMG, ‘Who invented this torture?’ Rather than a long lesson from Adrian's invention of the concentric needle onwards, we just say many contributed from the middle of the last century onwards. We may mention advances in the equipment, manipulation of images, averaging etc., though in truth there have been relatively few advances in clinical practice, say single fiber EMG, and quantitative studies. It appears that the basics of peripheral electrodiagnostics have remained relatively constant.

This rather stolid and unspectacular view, however, may be wrong. In the present volume of the IFCN's Handbook of Clinical Neurophysiology, the third, experts consider where their subject may be in five or more years' time, with the present chapter being on the future of peripheral neurophysiological tests.

It begins with needle EMG and motor unit number estimation (MUNE), followed by surface EMG and movement analysis. The next contribution surveys the possible impact of non-invasive magneto-neurography (MNG) and magneto-myography (MMG). We have known magnetoencephalography for decades now; how useful might the technique be applied to nerve and muscle?

The next two sections focus on electrical impedance tomography (EIT) and electrical impedance myography (EIM), which relate to peripheral nerves and muscles. In these, painless weak high frequency currents are used to investigate the passive electrical properties of nerve and muscle. They may provide complementary data to the active electrical properties of tissues usually recorded.

A section on microneurography details combining microneurographic analysis of single nerve cells' electrical properties with single-cell RNA-sequencing of nerve cells taken from dorsal root or trigeminal ganglia from deceased human donors. This allows a cell's ion channels to be related to its adequate peripheral stimuli.

The future clinical utility of excitability testing in nerve and muscle is then reviewed. The more recent use of excitability testing in muscles may assist the diagnosis of critical illness myopathy, muscle channelopathies and diseases associated with muscle hyperexcitability.

The last section considers ultrasound. Arguably, clinical neurophysiology was a slow adopter of this technique compared with rheumatology and radiology, though now it is now a routine part of the clinical neurophysiological evaluation of common neurological conditions in many departments. Various types are detailed with uses in different tissues and conditions.

These ten sections move from the enhancing of established techniques to consideration of novel research methods, some with clinical potential, others likely to be of academic interest. All show that the future is full of promise for peripheral clinical neurophysiology, whether through AI enhancing diagnostic accuracy of established tests, or through the application of new techniques.

## 2 Novel approaches to needle EMG and nerve conduction studies

Conventional needle EMG detects spontaneous activity indicating denervation, such as fibrillation potentials, positive sharp waves, and fasciculations at rest. During slight contraction, motor unit potential (MUP) characteristics, i.e. amplitude, shape, and stability are assessed while with full contraction, the interference pattern can evaluate motor unit recruitment and density.

MUP and interference pattern analyses can detect reinnervation and distinguish neurogenic from myopathic disorders. Qualitative assessment is more common, though it is subjective and depends heavily on the examiner's experience. Quantitative analysis is more accurate and objective but more time-consuming. However, computer-assisted quantitative EMG analysis programs (Bischoff et al., 1994; Nandedkar et al., 1995) and decomposition techniques (Stashuk, 1999) have reduced the time required.

Decomposition-based EMG also includes advanced near-fiber EMG algorithms. These use standard EMG recordings during low-to-moderate muscle contractions to enable semi-automated evaluation of electrophysiological properties including instability of motor units (Piasecki et al., 2021). Near-fiber EMG-derived measures of motor unit characteristics (e.g., duration, dispersion, and peak count) and instability (e.g., segment jitter) correlate with electrophysiological changes such as muscle fiber loss, atrophy or hypertrophy, axonal sprouting, neuromuscular junction formation, variability in neuromuscular junction transmission timing, and transmission failure. Near-fiber EMG complements traditional measures of MUP complexity, e.g., number of turns or phases and instability (e.g., jiggle) and has been used to demonstrate the effects of various neuromuscular disorders on individual motor units (Debenham et al., 2025; Garnes-Camarena et al., 2025).

Advances in AI and statistical methods may enhance quantitative EMG, while machine learning algorithms have already been used to identify neuropathic and myopathic patterns, and reduce variability in MUP analysis (Taha et al., 2026; Taha and Morren, 2024; Tannemaat et al., 2023). Recently, automated interference pattern analyses have been proposed, termed ‘filling analysis,’(Mariscal et al., 2025) and in a separate study, an ‘augmented intelligence’ algorithm has been developed which mimics subjective assessment (Nandedkar and Barkhaus, 2025). Another interesting development in statistical methods is the establishment of reference values, such as *E*-norms (Jabre et al., 2015). E-norm techniques are applied to MUP and NCS, and can be applied to individual laboratories (Jabre et al., 2015; Dunker et al., 2024). Others have used AI-driven NCS models to improve diagnostic accuracy and even predict diagnoses (Martinez-Thompson et al., 2025). AI may refine EMG and NCS analysis and enable analysis between labs and between experts, which thus far has proved difficult.

## 3 Motor unit number estimation (MUNE) methods

### 3.1 Introduction and evolution of MUNE methods

One of the most informative measurements in conventional NCS is the compound muscle action potential (CMAP). But, while a reduced CMAP amplitude can indicate axonal loss, as a result of collateral reinnervation, CMAP amplitude may remain relatively preserved until more than 50% of axons are lost. To overcome this limitation, various MUNE techniques have been developed, starting with incremental stimulation MUNE (McComas et al., 1971) which measures stepwise CMAP increases with increasing stimulus intensity. There are, however, several problems inherent in the method. Thresholds of units may overlap, so their recruitment alternates, generating more steps than units. To avoid this multipoint stimulation MUNE (MPS-MUNE) method was introduced, in which single surface MUPs are elicited by stimulating the nerve at multiple locations along its course (Kadrie et al., 1976). Like incremental MUNE, however, MPS-MUNE values are biased by the sampling of more units with the lowest electrical thresholds, and phase cancellation has been a major limitation of this method. To reduce these limitations, these two techniques have been combined in the so-called adaptive MPS-MUNE, in which incremental stimulation is applied at more than one site, however, its use has been limited to only a few studies (de Carvalho et al., 2018). Later, the spike-triggered averaging (STA) MUNE technique was introduced, which requires needle EMG to sample individual MUPs (Bromberg, 1993). This method is limited not only by the discomfort of needle insertion but also by the need of patient cooperation. A further selection bias occurs with the use of F-wave amplitudes to estimate average surface MUPs, on the assumption that repeated identical F-waves represent single units (Stashuk et al., 1994). In recent years, automated F-wave MUNE methods have been introduced (Artug, 2023). However, F-waves are influenced not only by peripheral motor nerve integrity but also by the excitability state of the spinal motoneuron pool, which is modulated by sensory input. Thus, surviving motor axons may still generate F-wave responses, and axonal loss may be overlooked, for example, in sensorimotor polyneuropathies (Yazici Gencdal et al., 2025).

More recently the motor unit number index (MUNIX) in which a motor unit number index is estimated from the surface EMG interference pattern (SIP) was introduced and incorporated into several EMG machines (Nandedkar et al., 2004). It is fast, typically under five minutes, and is tolerated well. It has been used as an outcome measure in several drug trials (de Carvalho et al., 2018; Lin et al., 2024).

### 3.2 MUNE methods based on CMAP scans

To avoid the selection bias inherent when using a limited selection of surface MUPs, MUNE can also be derived from a ‘CMAP scan’. These are detailed stimulus-response curves after stimulating a nerve and plotting CMAP amplitudes against stimulus intensity (Blok et al., 2007). These can use either exponential (% change in stimulus intensity) or linear (a fixed number of stimuli at increasing or decreasing intensities) protocols and allow visual and semiquantitative estimates of motor neuron loss (Blok et al., 2007). The method is generally well-tolerated, though it does require a large number of strong stimulations, and causes mild to moderate discomfort, even in lower extremity nerves (Tankisi et al., 2022). In healthy individuals, the CMAP scan typically displays an S-shaped curve, whereas in disease this pattern shifts to a stepwise form (Fig. 1) which enables various semi-quantitative parameters to be measured and has been proposed as a sensitive marker of amyotrophic lateral sclerosis (ALS) (Sleutjes et al., 2014). Despite its potential, it has not gained widespread adoption.

**Fig. 1:**
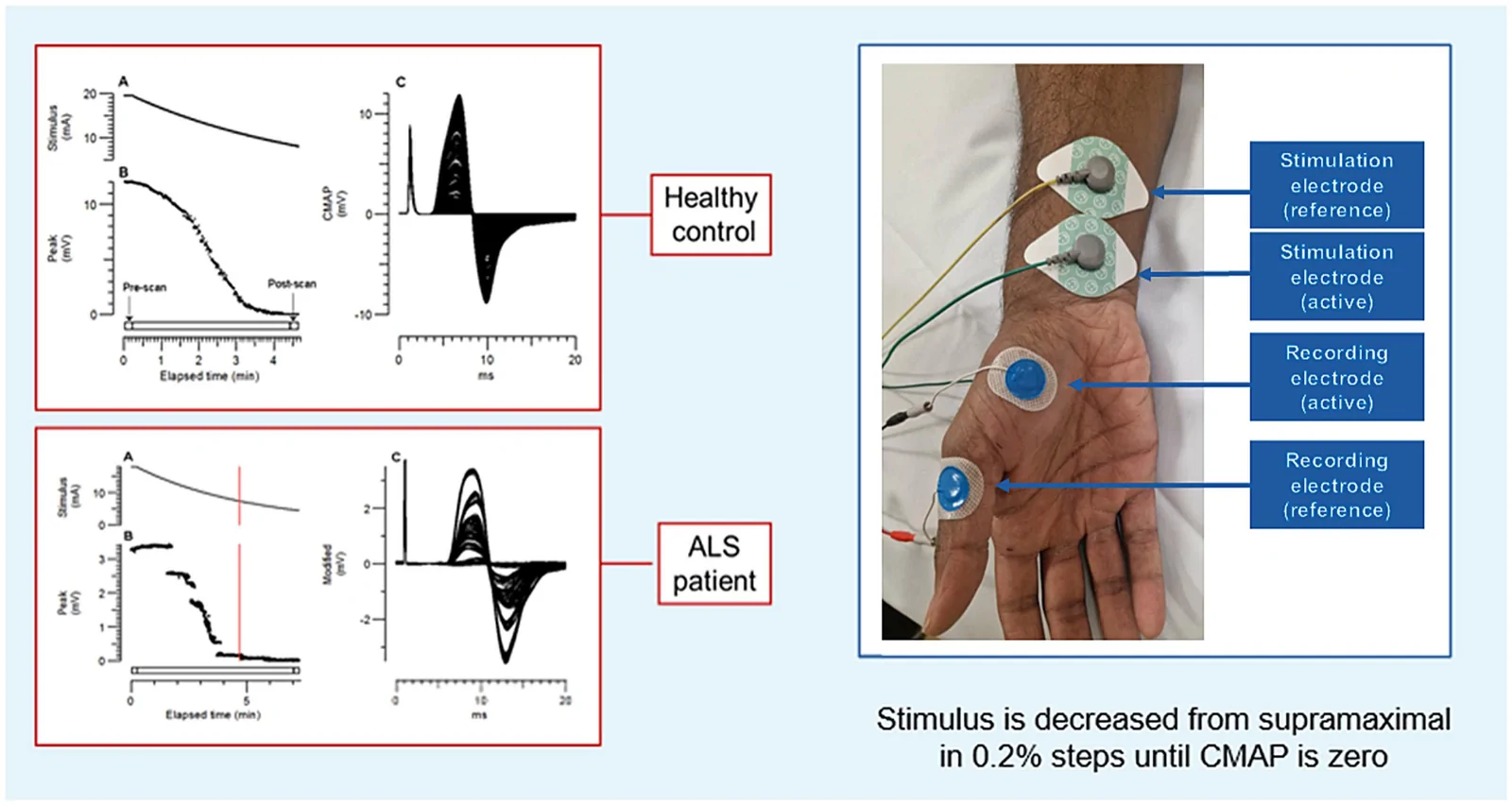
Examples of compound muscle action potential (CMAP) scans from a healthy subject and a patient with amyotrophic lateral sclerosis (left). For a median nerve CMAP scan, the stimulation electrodes are placed at the wrist and the recording electrodes on the thenar muscles (right).

The first MUNE method to take account of threshold variability and to estimate the thresholds and amplitudes of all the units contributing to a CMAP scan was the ‘Bayesian’ statistical approach (Henderson et al., 2007; Ridall et al., 2006). However, this mathematically sophisticated approach has not been widely adopted, since the analysis requires special software and can take several hours (Drovandi and Pettitt, 2013). A much quicker method, involving several simplifying assumptions, is ***MScanFit MUNE*** (Bostock, 2016). This is a two-step method: first, a preliminary model is created based on the slope and variance of the responses relative to stimulus intensity in the main scan. This model is then progressively refined by adjusting individual motor unit parameters to reduce the ‘error’ (a measure of the difference between the recorded scan and the modelled one) (Fig. 2).

**Fig. 2:**
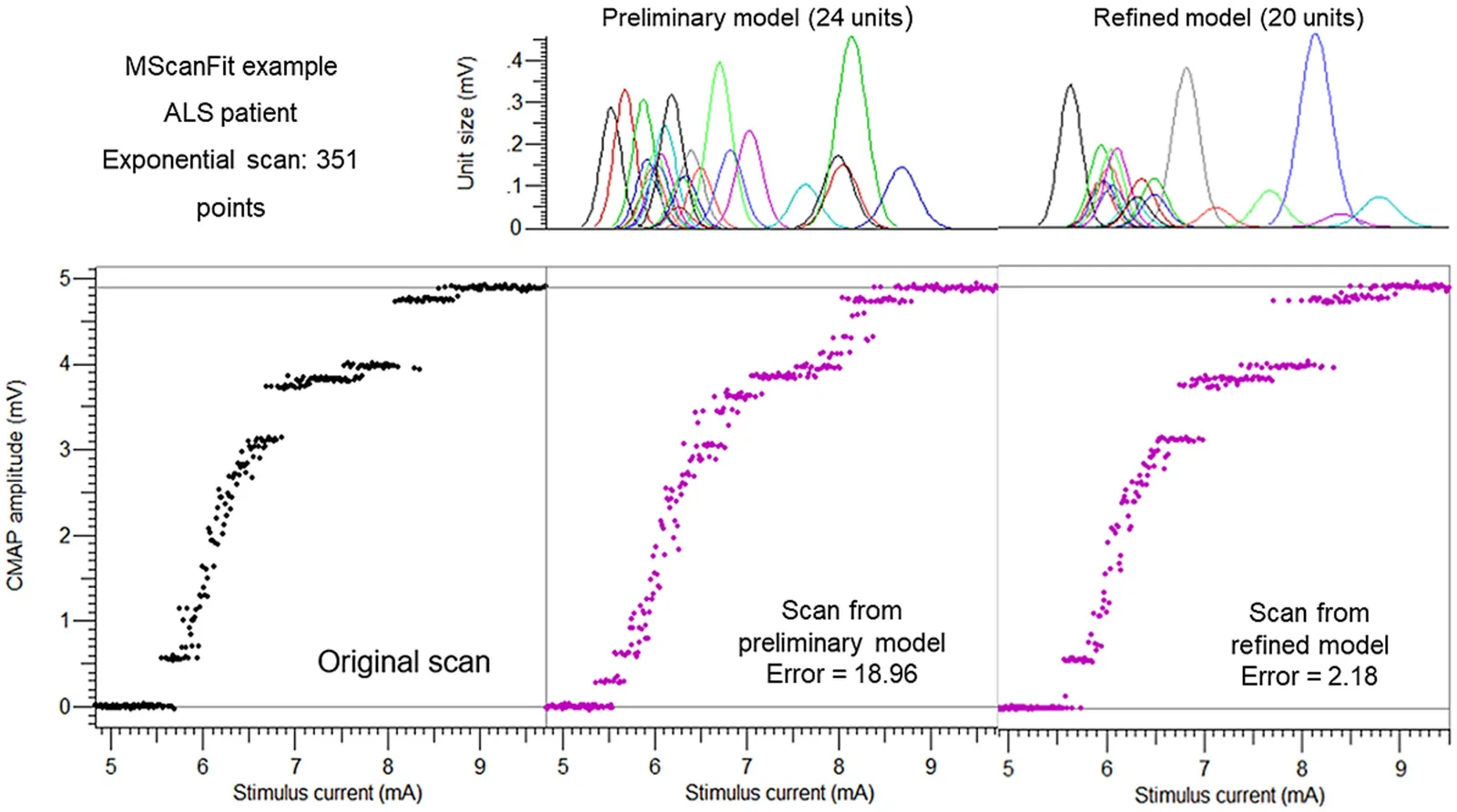
An example of MScanFit method of motor unit number estimation (MUNE). On the left is the original compound muscle action potential (CMAP) scan or detailed stimulus-response curve. The middle panel shows a preliminary model, derived from the slope and variance of segments of the original scan. At the top each unit is represented by a normal curve, the peak height corresponding to the unit amplitude, and the width indicating the threshold variability. The panels on the right indicate the refined model, after the unit properties had been progressively adjusted to minimise the differences from the original scan.

MScanFit was released as a freeware program, but has most often been used as part of the specialized QtracW© software (copyright Institute of Neurology UCL, London, distributed by Digitimer Ltd., www.digitimer.com) which enables exponential scans. It is non-invasive, and easy and fast to perform, with an automated analysis. The technique is promising as a diagnostic tool or an endpoint measure in clinical drug trials. MScanFit has been utilised in a variety of diseases, including ALS (Jacobsen et al., 2017; Jacobsen et al., 2019; Kristensen et al., 2019b; Stikvoort Garcia et al., 2023), diabetic polyneuropathy (Kristensen et al., 2019a) and spinal muscular atrophy (Vacchiano et al., 2024). In these studies, MScanFit has shown greater sensitivity to detecting motor unit loss than conventional electrophysiological tests and clinical scores.

More recent approaches to obtaining MUNE from CMAP scans are ***STEPIX*** (number of ‘steps’), which uses response amplitudes only(Nandedkar et al., 2022), ***STAIRFIT,*** which fits a staircase function (Chen et al., 2023) and ***CDIX*** (CMAP distribution index), based on calculation of the information entropy (Lu et al., 2023). It remains to be seen which of these four MUNE methods based on CMAP scans proves most useful diagnostically and as outcome measures in drug trials.

## 4 Surface EMG and analysis of movement

### 4.1 Introduction

Recording muscle activity is an essential aspect of clinical neurophysiology. This involves the steps described in the process of excitation-contraction coupling (Fig. 3): recording electrical currents travelling in muscle fiber membranes, i.e. pre-activity, and recording the mechanical effect of muscle contraction, i.e., the actual movement or force. Another step would be to record the consequences of muscle activity, i.e., task performance, which could be added for a complete functional assessment. The different equipment needed for each of these phases will be summarized, together with their clinical utility, with their future perspectives.

**Fig. 3:**
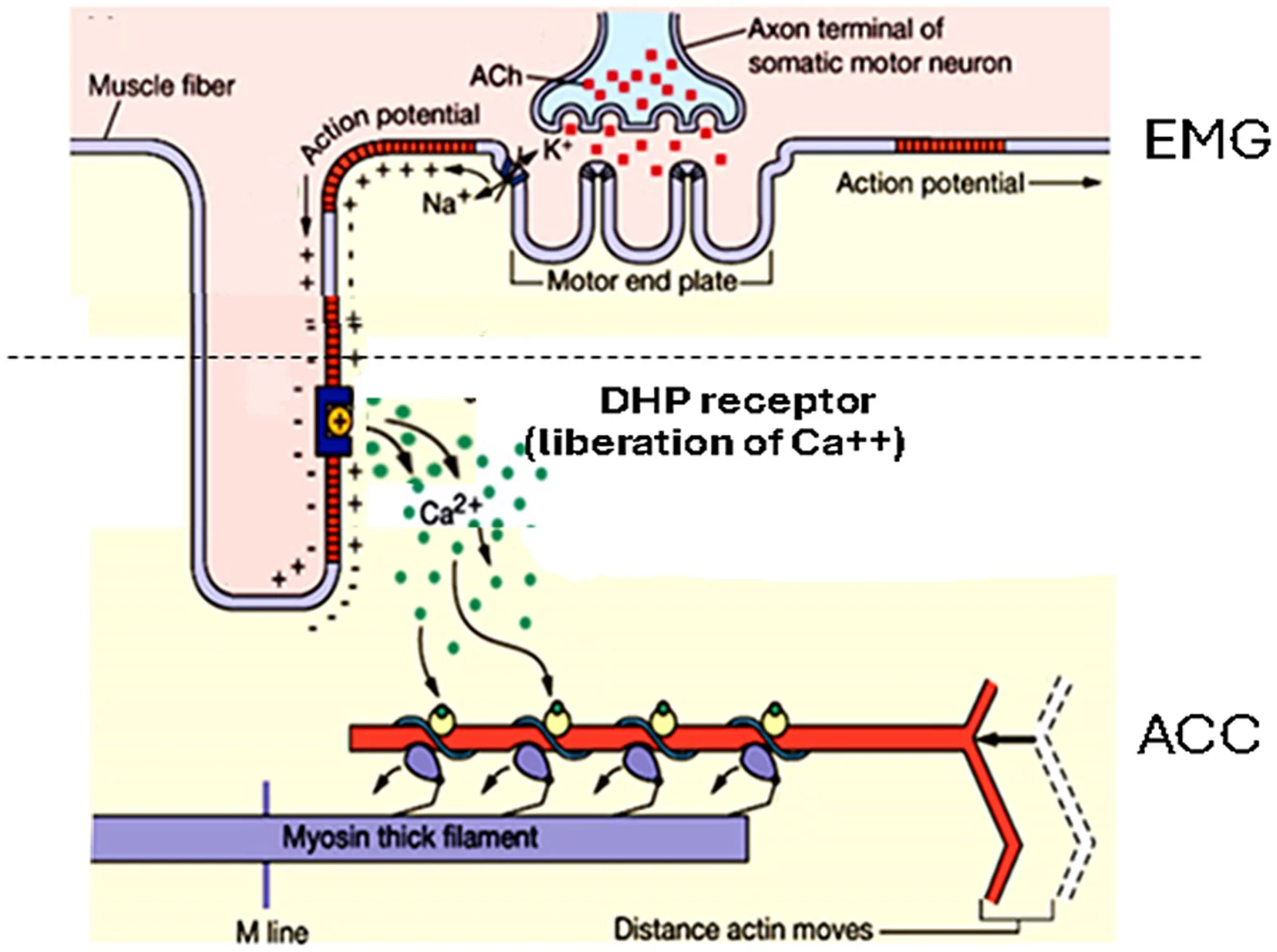
Schematic illustration of the two steps in the production of muscle activity: The upper part shows the arrival of nerve action potentials to the endplate and their propagation along the muscle fiber, which can be recorded using EMG electrodes. The lower part shows calcium release into the sarcoplasm inducing the mechanical process of sliding actin over myosin filaments, a mechanical process which may be recorded with movement or force transducers.

Surface electromyography (sEMG) records electrical currents in muscle fiber membranes through electrodes attached to the skin, usually with the active one near the muscle innervation zone and the reference at the tendon or a few centimeters apart (bipolar montage). It is more suitable than needle EMG for functional assessment of movements.

In high-density sEMG (Merletti et al., 2008), a grid of 8 to 64 electrodes is used to record the propagation of the muscle fiber action potential after its generation at the neuromuscular junction and as motor unit action potentials propagate from the innervation zone. This allows the velocity of propagation of action potentials in a motor unit to be calculated (Valli et al., 2024). Though this is useful in motor unit physiology, clinical applications are, as yet, scarce.

### 4.2 Movement and force transducers

Electrical activity in muscle fibers induces muscle contraction, mediated through an influx of Ca++ which binds to troponin and reduces muscle sarcomeres' length by sliding the actin filaments toward the myosin filaments. This shortening of muscle fibers can be recorded with muscle ultrasound (Hodges et al., 2003) or functional MRI (Hooijmans et al., 2024). Though these techniques can be useful in the diagnosis of certain neuromuscular diseases such as motor neuron disorders (van Alfen et al., 2018), they are of little use in the assessment of movement.

The result of muscle contraction is either force (isometric contractions) or movement (isotonic contractions). Both can be recorded with transducers that transduce energy from force (for instance, dynamometers) or movement (for instance, accelerometers) into electrical signals. Though the simplest movement transducer, a single-axis accelerometer, is usually adequate for clinical practice, more sophisticated triple axis accelerometers are readily available now on most mobile phones.

It is important to realise that the properties of muscle contribute to movement performance. Sarcomeres contribute to the generation of force or movement through their length-tension relationship (Rothwell, 1994), while a muscle's viscosity, elasticity, inertia, capacitance and other factors are integrated by the nervous system moment by moment for movement performance, whether walking, jumping, catching a flying object, or anticipatory postural activity accompanying an intended movement.

### 4.3 Signal analysis and methods useful in clinical practice

Although raw sEMG and accelerometer (ACC) recordings are enough for many purposes, quantitation of activity requires signal analysis. The most common sEMG signal analysis procedure is rectification (Fig. 4). Averaging rectified epochs of sEMG during a sustained contraction leads to a relatively smooth line showing mean EMG activity, essential for quantitation of inhibitory effects of sensory inputs (Kofler et al., 2019). The same procedure is used for mathematical integral or the root-mean-square value, a quantitation of sEMG activity used for many purposes (Khaliliyan et al., 2024; Silva Junior et al., 2023).

**Fig. 4:**
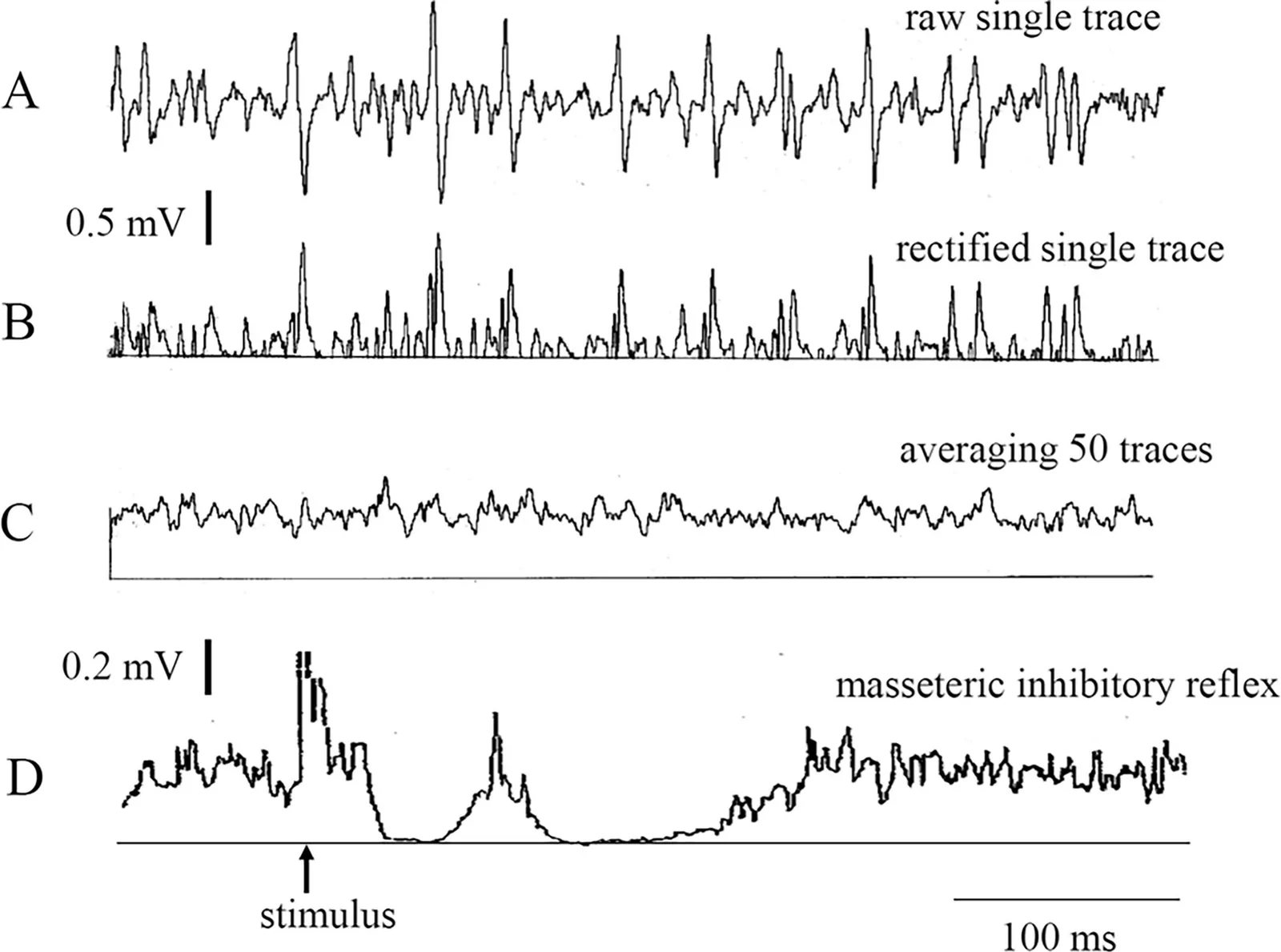
Procedure for averaging rectified surface EMG (sEMG) traces during a mild contraction: Trace A shows a single EMG epoch of 500 ms duration, with its characteristic upgoing and downgoing waveforms. Trace B shows the same epoch rectified, i.e., with all waveforms upgoing. Trace C shows the result of averaging 50 rectified epochs, which led to a relatively smooth line at a mean level above baseline. Trace D shows the result of applying this technique to record the masseteric inhibitory reflex. The stimulus was applied at the arrow.

Reaction times are frequently used in the assessment of human movements. Simple reaction time tasks require subjects to perform a well-defined movement as fast as possible after the imperative signal. sEMG records the so-called pre-movement reaction time, while an ACC placed over the moving segment records the movement, and a transducer records task accomplishment. A controlled ballistic movement, like wrist extension, performed in a simple reaction time task gives rise to the so-called triphasic pattern (Hallett et al., 1975), which is considered the most basic motor program.

When the prime mover in a reaction time task is pre-activated, maintaining a tonic contraction, the first burst of EMG activity is preceded by a pre-movement silent period, when motor units are quiescent before their synchronous recruitment in the contraction, see the soleus muscle in the tip-toeing maneuver (Fig. 5). Unilateral ballistic movements interfere with voluntary actions being performed with the contralateral hand.

**Fig. 5:**
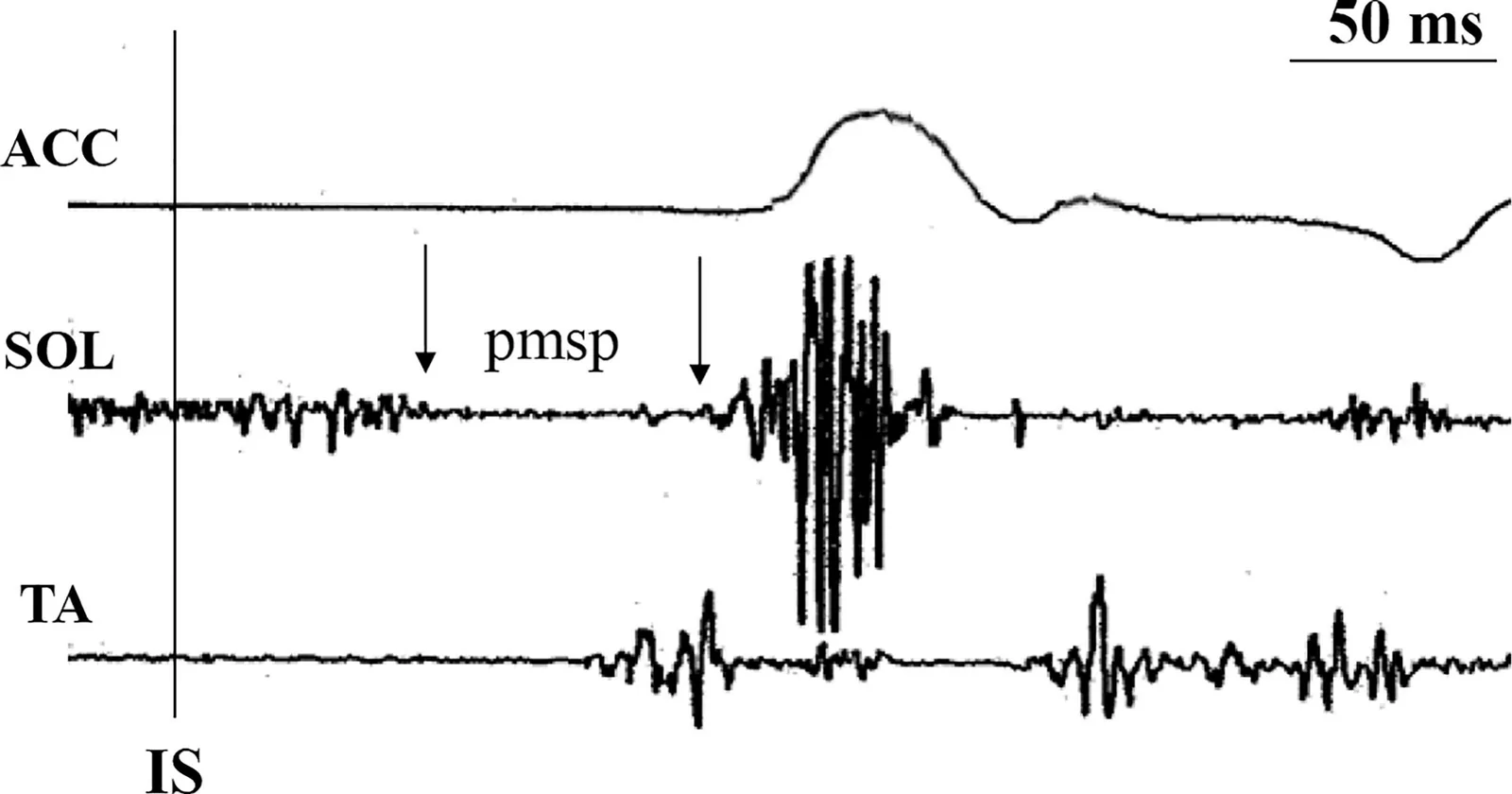
Recording the tip-toeing maneuver with an accelerometer (ACC) placed on the head and surface EMG electrodes placed on the soleus (SOL) and tibialis anterior (TA). A standing still subject was instructed to raise on tiptoes at the imperative signal (IS). Notice the arrest of tonic activity in the SOL indicated as the premovement silent period (pmsp), before a big phasic burst leads to the movement picked up by the ACC. The presence of some bursts in the TA may contribute to the transient inhibition of the SOL motoneurons.

More complex reaction times involve choice of reaction and Go-no Go reaction time tasks, which require an element of inhibition to suppress the undesired activity if the noGo signal appears. Though simple, reaction times have proved informative in some movement disorders (Hallett, 1990).

### 4.4 sEMG activity and ACC for the study of human physiology and neurological disorders

There is a large literature on the study of activities of daily living in health and disease including gait, standing balance, postural adjustments, and manual activities in which sEMG and ACC are used. Postural changes accompanying voluntary movements, essential for balance control, are for instance impaired in Parkinson's disease (Nardone and Schieppati, 1988). They can also be used in studies of peripheral fatigue (Tankisi et al., 2024b), when there is slowing of action potential propagation in the muscle fiber membrane, just before force begins to decrease.

sEMG and ACC are also useful in the assessment of hyperkinetic movement disorders (Apartis, 2013; Chen et al., 2022; van der Stouwe et al., 2021), functional movement disorders (Edwards et al., 2023; Kamble and Pal, 2023), and are essential for the classification of tremors (Deuschl, 1999). Fast Fourier transformation of the ACC signal helps in differentiating between tremor and other forms of hyperkinetic disorders such as myoclonus or dystonia (Erro et al., 2015; Salazar et al., 2000). In orthostatic tremor, ACC recordings may show little, while sEMG from leg muscles show the typical high frequency of tremor bursts and a characteristic sound. In functional tremor, entrainment by asking patients to perform a rhythmic action with a different limb, and ballistic suppression in a reaction time task, are, among others, key supports to clinical diagnosis (Schwingenschuh and Espay, 2022).

sEMG and movement transducers may also help in devising better prostheses in neurorehabilitation. The burst of sEMG activity that patients may still produce in some muscles may serve as feedback for selective neuromuscular electrical stimulation (Balbinot et al., 2022; Knutson et al., 2015) or activation of electronically driven prosthetic devices (Won and Iwase, 2024). However, we are still far from a satisfactory control of prostheses. Ideally, they should imitate physiological function, an aim needing teams of experts including neurophysiologists.

### 4.5 Automated assessment and projection to the future

Technology and clinical expertise will continue to improve the quality of life for neurological patients. Technological advances have often gone hand in hand with development of neurophysiological methods used in research and clinical practice. This will continue as complexity increases in the era of robotics and AI. sEMG is likely to remain the best basic signal for successful control of robotic systems because of its non-invasiveness and ease of recording. The combination between sEMG and other biological signals, whether neurophysiological (EEG, autonomic reactions, force, movement) or other (clinical, radiological, biological) will allow further development and help define algorithms for real time control of prosthetic devices. Brain-machine interfaces may be improved by incorporating into their algorithm brain-muscle features, like cortico-muscular coherence, to improve the accuracy of machine-driven movements (de Seta et al., 2024).

Algorithms for diagnosis and prognosis are also being developed by feeding the system with a list of expert-based classifiers. van der Stouwe et al. (van der Stouwe et al., 2021) are engaged in an ambitious project to improve the classification of hyperkinetic movement disorders in this way. The authors obtain data from sEMG, ACC and video recordings in patients with hyperkinetic movement disorders, including myoclonus, dystonia, tremor, chorea, tics, ataxia, spasticity and functional movement disorders to feed a machine learning algorithm. It is hoped the algorithm may predict the diagnosis when given new input data. sEMG-based machine-learning has been also used for the assessment of balance disorders in patients with PD (Castelli Gattinara Di Zubiena et al., 2022), tremor (Saad et al., 2024), sleep (Balachandar et al., 2024) and other disorders. Technological developments may bring new methods and techniques in the near future, with the aim of overcoming the limitations of sEMG and ACC in the control of prosthesis and robots. sEMG and ACC use may benefit from improved feature extraction techniques beyond the conventional threshold and amount of sEMG activity. Principal component analysis and time-frequency domains are being investigated, and they may likely improve the existing technologies in clinical, neurophysiological, and rehabilitation domains (Yadav and Veer, 2023).

Alternative methods to sEMG include muscle ultrasound and force myography. In both cases, the rationale is to use muscle contraction as the input for machine-learning (Nazari and Zheng, 2025). In one study, the residual force generated by voluntary muscle contraction was used to interpret the patient's intention and trigger the appropriate robotic assistance (Gantenbein et al., 2023).

One requirement is to make any recording device user friendly and wearable, i.e., neither heavy nor large. Though wireless EMG recording systems have been around for some time (Sabino et al., 2025; Zhang et al., 2025), we are far from full implementation of them in the clinical context. The use of smartphones as diagnostic machines for various purposes may not be far away. They allow simultaneously recording bioelectrical signals and video images. There already are publications about designed mobile applications integrating data from independent wearable sensors, including sEMG, that are easy to use and do not interfere with daily work activities (Sabino et al., 2025).

Steps into a future, AI-loaded, clinical neurophysiology world must be taken carefully. AI-based research guidelines are needed to facilitate clinical utility (Vizcarra et al., 2024). sEMG and ACC recordings still depend on human factors such as the choice of the recording site, positioning of recording electrodes, identification of artifacts, and selection of suitable recording epochs, etc. Will new technologies be free of human intervention and potential errors? Seemingly trivial errors could have important consequences. As stated by Husain (Husain, 2025), we must establish clear rules for the use of AI to ensure that it augments rather than replaces human judgment in clinical neurophysiology.

## 5 Magnetoneurography (MNG) and magnetomyography (MMG)

Magnetoencephalography and magnetocardiography, which detect magnetic fields from the brain and heart respectively, are well established in research and clinical settings. In contrast, progress in MNG and MMG, measuring magnetic fields from peripheral nerves and muscle fibers, has been comparatively slow due to technical, biophysical, and practical challenges.

One challenge is that peripheral nerve and muscle magnetic fields are significantly weaker than those from the brain or heart, so sensors with extremely high sensitivity and spatial resolution are needed (Murzin et al., 2020). Another reason for the slower progress may be utility. While magnetoencephalography and magnetocardiography address well-established clinical needs such as epilepsy surgery planning and arrhythmia diagnosis, MNG's possible clinical applications are currently addressed satisfactorily by conventional NCS and EMG. This section will address MNG and MMG's applications that may increase their use.

### 5.1 Advantages and disadvantages of magnetoneurography and magnetomyography

In contrast to NCS and needle EMG, MNG and MMG are non-invasive and contact-free, requiring no skin electrodes. Since magnetic fields are not distorted by tissue in the way electrical signals are, MNG and MMG also offer higher spatial resolution compared to NCS and EMG. Additionally, magnetic fields penetrate tissue more effectively than surface electrodes, enabling the detection of deeper signals (Murzin et al., 2020). This is particularly advantageous for assessing structures such as nerve plexuses, nerve roots, the spinal cord, and deeply located muscles. Furthermore, MNG and MMG provide 3D spatial information, unlike electrical signals (Adachi and Kawabata, 2024; Noury et al., 2025).

The most important limitations of MNG and MMG are their need for expensive sensors and their complexity which requires advanced data processing and interpretation (Murzin et al., 2020). Indeed, these disadvantages have limited its used to research even though the technique has been available for several years since the first MNG was reported in 1980 (Wikswo et al., 1980), with a superconducting quantum interference device (SQUID) to record neural activity of a frog sciatic nerve. SQUIDs sensors have ultra-high sensitivity (∼1 fT/√Hz) but require cryogenic cooling with liquid helium, fixed sensor array geometries and a magnetically shielded room, making them expensive, large and immobile, and impractical outside of high-end labs. These technical constraints have been circumvented by recent advances in quantum sensing with optically pumped magnetometers (OPMs), which can be flexibly placed around body parts and muscles due to their miniaturized design and operation at room temperature, discarding the need for cryogenic cooling. Though slightly less sensitive than SQUIDs (∼10–100 fT/√Hz) they are also far cheaper. OPMs appear to offer a balance of performance and practicality to enable the transition of MNG and MMG from experimental to practical settings. Giant magnetoresistance and tunnel magnetoresistance sensors are more futuristic or in development, with potential for miniaturized or wearable systems (Murzin et al., 2020).

### 5.2 Applications of magnetoneurography

To date, induced magnetic fields have been measured from nerves in the carpal tunnel (Sasaki et al., 2020; Sato et al., 2023), elbow (Tanaka et al., 2024), upper arm (Nakayama et al., 2023), and brachial plexus (Ozaki et al., 2023; Watanabe et al., 2019). These studies are based on peripheral nerve stimulation at the digital nerves and of the median and/or ulnar nerves at the wrist or elbow, with results compared to conventional NCS. Peak latencies have shown comparable results. Studies in patients have also been reported (Sasaki et al., 2022; Sasaki et al., 2020). In patients with carpal tunnel syndrome (CTS), the injury sites, undetectable by NCS, can be identified using MNG's higher spatial resolution (Sasaki et al., 2022).

MNG has also been applied at the cervical spinal cord, the so-called magnetospinogram after stimulation of peripheral nerve, i.e. median nerve at the wrist (Akaza et al., 2021). A peak in the magnetic wave was seen at C5 with the same latency as the N13–P13 electrical potential. MNG may allow the examination of conductive disorders in the dorsal column and record activity in the dorsal horn, showing the origin of different somatosensory evoked potential components including P9 (Paku et al., 2023), again using its higher spatial resolution.

### 5.3 Applications of magnetomyography

MMG studies of human muscles have recently gained attention (Baier et al., 2025; Broser et al., 2018; Ghahremani Arekhloo et al., 2023; Klotz et al., 2023; Nordenstrom et al., 2024). An in-silico simulation study found that high-density MMG detected 76% more motor units than high-density surface EMG. Moreover, MMG showed less bias toward superficial motor units, providing a more balanced representation across depths in the tissue (Klotz et al., 2023). A proof-of-concept study showed that non-invasive MMG could detect the same motor unit discharges captured invasively by integrated EMG, offering strong preliminary validation of MMG's potential as a non-contact alternative (Broser et al., 2021). In a recent study, individual motor unit activity was accessible using non-contact MMG. Future research on motor unit decomposition may use MMG to study muscle activity in health and disease (Noury et al., 2025).

In summary, MMG may detect motor unit activity comparably to invasive EMG. However, most research so far is simulation-driven or proof-of-concept. Clinical validation and routine applications are still under exploration.

## 6 Electrical impedance tomography (EIT)

### 6.1 Introduction and current state of the art

EIT is a comparatively new technique which enables images of the internal impedance of the subject using a small, insensible electric current – typically about 1 mA at 10 to 50 kHz – applied sequentially to multiple pairs of electrodes. Images are reconstructed from the resulting measurements of transfer impedances made by recording voltages at the other electrodes. The images have a similar resolution to those obtained with radioisotopes in PET or SPECT, and are slightly blurred. Images may be collected rapidly, up to hundreds per second using a box of electronics about the size of an EEG machine, a computer and with an array of EEG or ECG electrodes placed around the body part of interest. The technique's principal research interest has been imaging lung ventilation. Although the images have lower resolution than MRI or CT, the technique is gaining acceptance as a portable bedside monitor of ventilation in patients on intensive-care units.

Holder and colleagues at UCL have adapted this technique for imaging small impedance changes of about 0.1% which occur in neural tissue when depolarising. Initially, their interest was to image fast neural circuit activity in the brain during normal activity and epilepsy. This was achieved in 2016, but only proved possible with arrays of subdural electrodes placed upon the surface of the cerebral cortex in experimental animals. It also required averaging a repeated stimulus over several minutes. With this approach, it was possible to produce images of neuronal depolarisation over milliseconds anywhere in the cerebral cortex to a depth of about one quarter of the brain with a resolution of 2 ms in time and 0.2 mm in space (Aristovich et al., 2016).

More recently interest has been in imaging compound action potentials in fascicles of the cervical vagus nerve. Electrical stimulation of the nerve in epilepsy and depression has unwanted side-effects, such as hoarseness due to inadvertent stimulation of the whole nerve, affecting fibers to the larynx. Fast neural EIT has the potential to image organ-specific function in internal fascicles in the nerve, enabling selective stimulation and so avoid side-effects. Organ-specific fascicles have been imaged in the cervical vagus nerve of pigs and sheep related to pulmonary, cardiac, or laryngeal function, with two rings of 14 electrodes placed surgically around the cervical vagus nerve and which were also used for selective neuromodulation of identified fascicles (Thompson et al., 2023).

Preliminary human studies show similar findings. Biomedical EIT is attracting international interest, especially in China, and there are now about ten companies developing EIT systems for imaging ventilation and cardiac output. Systems for imaging brain or nerve function remain at the development phase.

Human studies so far have exclusively been on the cervical vagus nerve, though the approach could be applied to studies of fascicular function in somatic peripheral nerves as well. The technology and nerve cuff have medical safety approval in the UK.

Fast neural EIT (fnEIT) enables tomographic imaging of localized compound action potential activity in fascicles within peripheral nerve. It uses a flexible silicone rubber cuff with 14 electrodes and averaging to a repeated trigger. A current of ∼50 μA at ∼6 kHz is successively applied to pairs of electrodes with decreases of ∼0.1% in resistance (Aristovich et al., 2014; Avery et al., 2017).

The method was initially validated in rat sciatic nerve. Its branches were electrically stimulated peripherally in the leg of anesthetized rats at 5 Hz and EIT images of fascicles obtained averaging over one minute from a silicone recording nerve cuff placed around the sciatic nerve in the proximal thigh. Separate fascicles could be imaged and correlated with independent measures from X-ray microCT and fluorescent dextran neural tracers (Ravagli et al., 2020). Large diameter myelinated fibers were imaged since these produce the largest impedance changes when depolarized during the action potential. The temporal resolution was similar to that of NCS, i.e. ms (Fig. 6) (Ravagli et al., 2020).

**Fig. 6:**
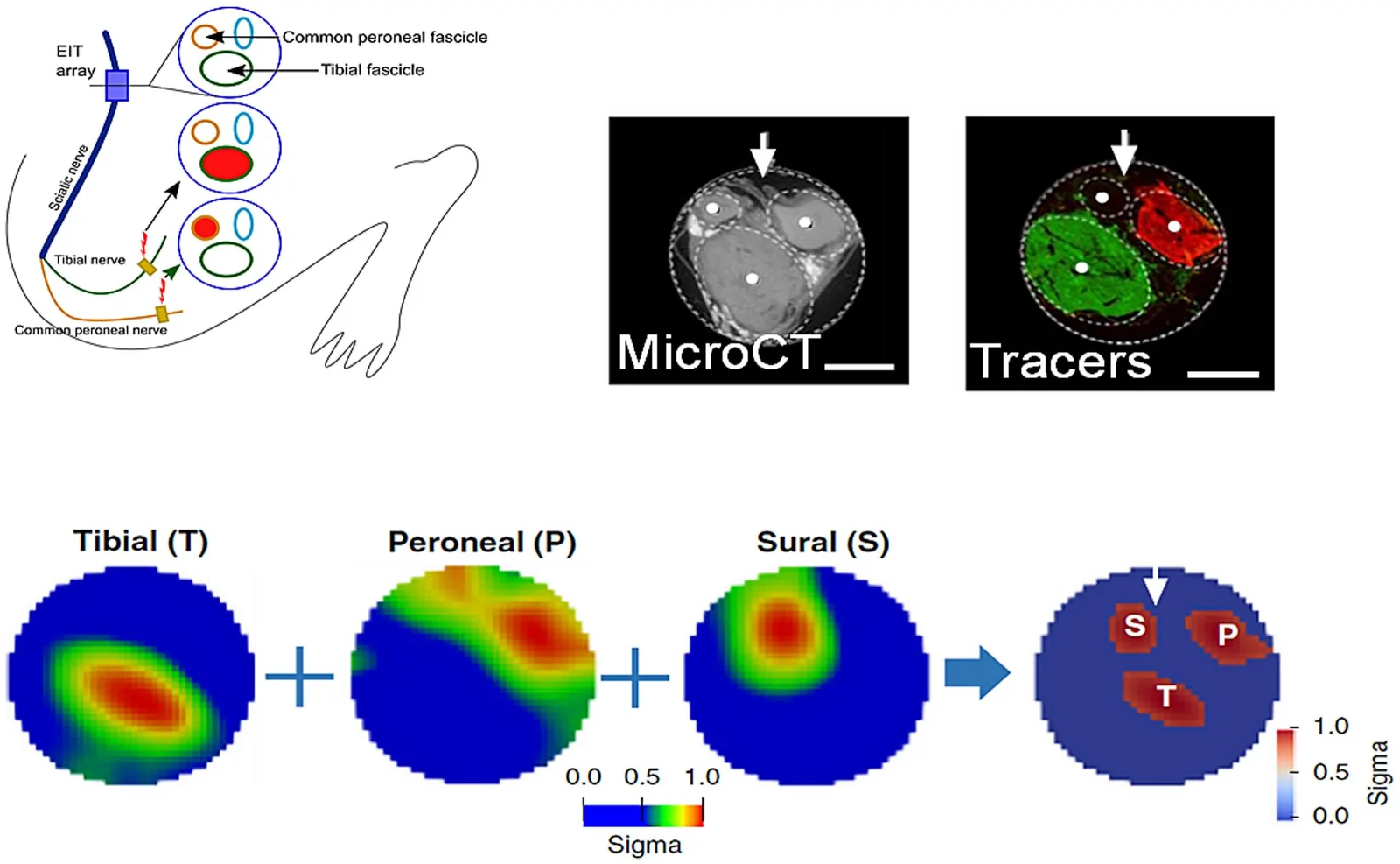
Images of fast neural electrical impedance tomography (EIT) in rat sciatic nerve (Ravagli et al., 2020).

The functional anatomy of fascicles in the cervical vagus nerve of anesthetized pigs has also been determined. fnEIT images were correlated with microCT in nerves excised along their full length from the vagus nerve in the neck to the target organs – heart, lungs and larynx (Thompson et al., 2020; Thompson et al., 2023). By serial selective stimulation, three spatially separated fascicular groups were shown, correlating with cardiac, pulmonary, and laryngeal activity. In cross-section, if recurrent laryngeal fibers are placed at the top (Fig. 7, Fig. 8).

**Fig. 7:**
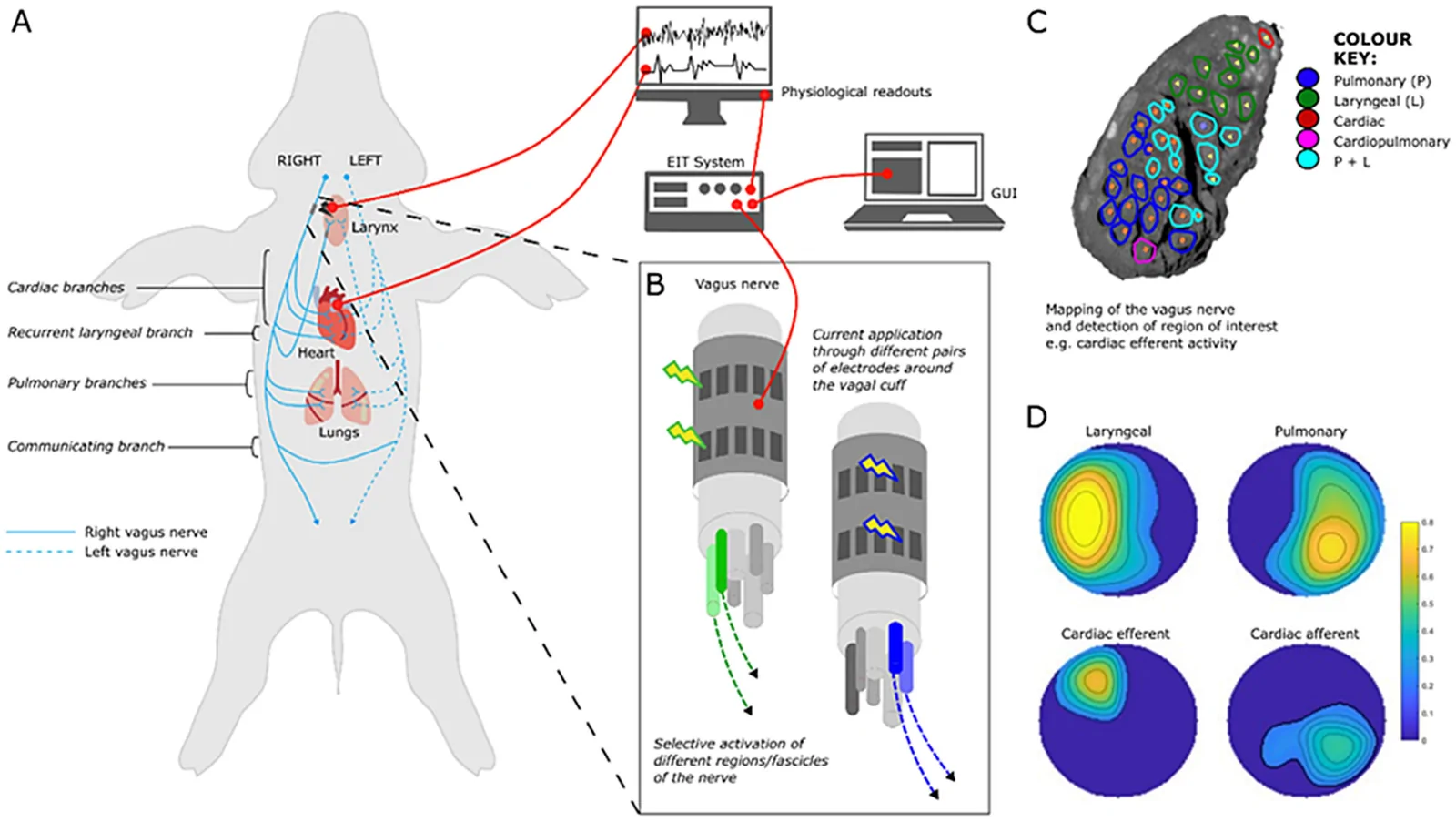
Pig studies identifying afferent and efferent cardiac localisation in the cross-section of the cervical vagus nerve structurally and functionally. (A-B) A multi-electrode cuff for selective vagal nerve stimulation. (C) The dissected right vagus of the pig was contrast-stained and microCT scanned and fascicles traced from their organ origin to the cervical level to produce a labelled and mapped cross-section at the mid-cervical level. (D) The mean regions comprising laryngeal, pulmonary, cardiac efferent and cardiac afferent fascicles or function (*n* = 10) following selective stimulation. The colour scale, from 0 to 1, depicts the proportion of fascicles or electrophysiological responses upon selective activation that were located within the area in the cross-section across all the nerves (0 indicates no fascicles were present at the location in any nerve, 1 indicates that all nerves had a specific fascicle in that location) (Thompson et al., 2023).

**Fig. 8:**
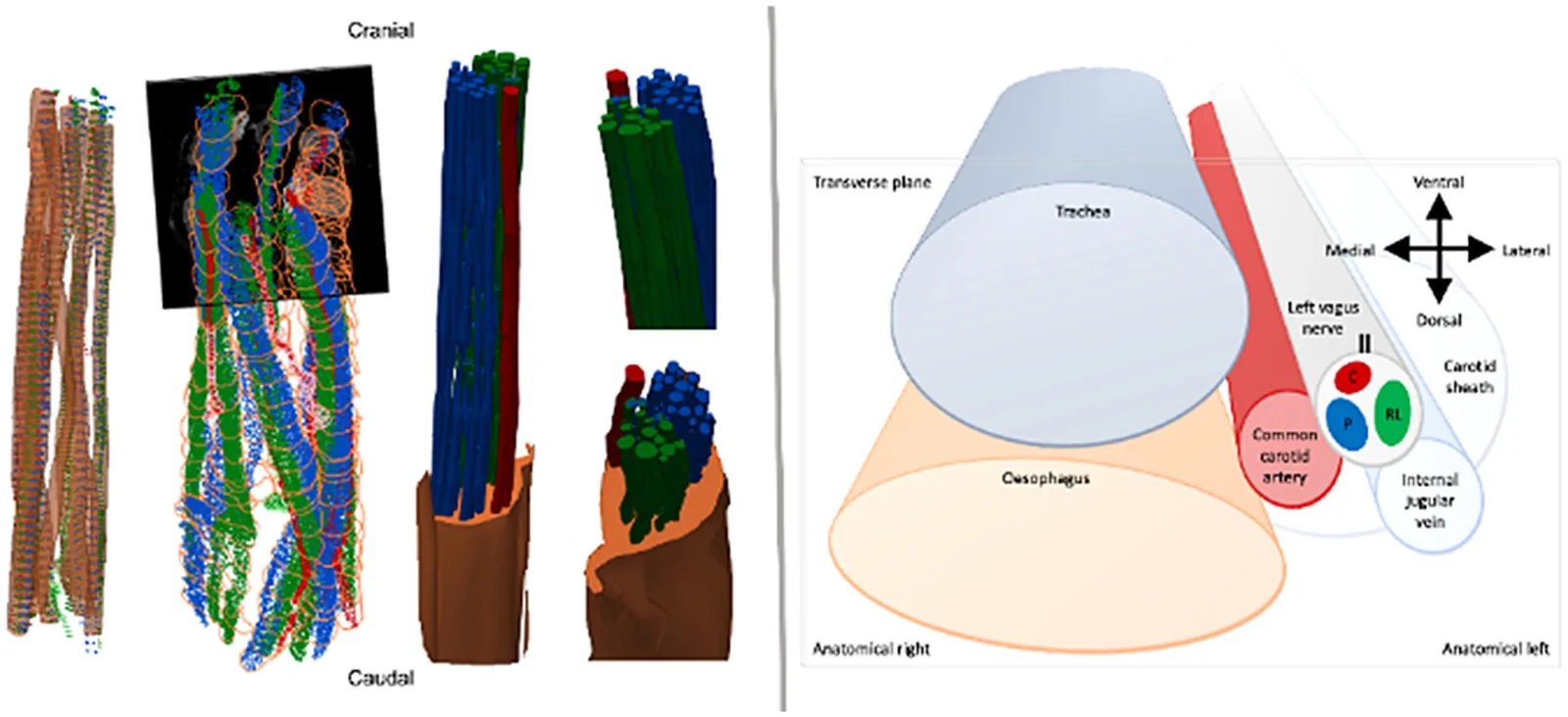
Confirmation of fast neural electrical impedance tomography (fnEIT) fnEIT image findings with fascicle tracing with microCT of iodine stained vagus nerves (*N* = 4) (laryngeal in green, pulmonary in blue and cardiac in red) in the anesthetized pig (Ravagli et al., 2023). Left: 3D shelled segmentation of fascicles in the nerve. Right: Schematic of anatomical location of fascicular regions of the left vagus nerve within the pig with a caudal to cranial view. The opening of the cuff is indicated with a double black line and the fascicular groups have a clockwise order of laryngeal, pulmonary and cardiac. V = ventral, VM = ventromedial, M = medial, DM = dorsomedial, D = dorsal, DL = dorsolateral, L = lateral, and VL = ventrolateral. (For interpretation of the references to colour in this figure legend, the reader is referred to the web version of this article.)

Human studies are in progress in patients with epilepsy undergoing surgical implantation of vagal nerve stimulators. Preliminary data in five subjects indicate similar anatomy with specific cardiac and pulmonary separated fascicles.

### 6.2 Future perspectives

fnEIT reveals the functional connections of nerve fascicles after selective stimulation. Though safe for human use, it requires cuffs to be surgically implanted around the nerve. A further limitation is that, currently, fnEIT images are only of large myelinated fibers in peripheral nerves, since impedance changes from small myelinated or unmyelinated fibers are smaller and show temporal dispersion, problems which may be overcome with signal processing and prolonged averaging (Tarotin et al., 2022).

At present, its principal application lies in neuromodulation of autonomic nerves. So far, electrical vagus nerve stimulation has received FDA approval, with unselective whole nerve stimulation, for the treatment of drug-resistant epilepsy, arthritis and depression. However, vagus nerve stimulation might be useful in the treatment of cardiovascular disorders and heart failure, lung injury, asthma, sepsis, rheumatoid arthritis, diabetes, obesity, pain management, and the reduction of autoimmunity via the anti-inflammatory pathway. Side effects due to inadvertent stimulation of the entire nerve include cough, dyspnoea and hoarseness, shortness of breath and bradycardia, and long-term side effects, such as the development of hyperglycaemia when stimulating to treat epilepsy. Mapping of the anatomy of fascicles and selective stimulation might reduce side effects but improve the efficacy of vagus nerve stimulation overall.

FnEIT may be useful in other areas. It could be used to identify dermatomal or myotomal fascicles, which might improve results of microsurgical fascicle repair. At present, production of fnEIT takes about a minute though real-time fnEIT images is being investigated. Then fnEIT could be used in the control of prosthetic robotic limbs, since it could enable real-time recording of multiple fascicle activity from intact proximal nerves.

## 7 Electrical impedance myography (EIM)

### 7.1 Introduction and state of the art

EIM uses painless, weak, high frequency electrical current applied to a region of muscle through two electrodes, with the consequent voltages measured through a second set of two electrodes (Rutkove and Sanchez, 2019). These are affected by the intrinsic properties of the muscle tissue, including its resistive elements (e.g. free water and fat) and its capacitive elements (mainly cell membranes, including the sarcolemma). This contrasts with normal neurophysiological methods for assessing nerve and muscle potentials through the active electrical properties of tissues. EIM provides data on the passive electrical properties of muscle, giving insight into the tissue's structure and composition, thus providing value as a potential primary diagnostic modality as well as tool for tracking disease progression or response to therapy in an efficient and convenient manner. EIM is often performed in the resting state, but use of EIM during active contraction can offer additional information on the muscle's condition, including, potentially, intrinsic aspects of the muscle contraction process itself (Xu et al., 2024). Fig. 9 provides an overview of approaches for performing EIM in humans and rodents.

**Fig. 9:**
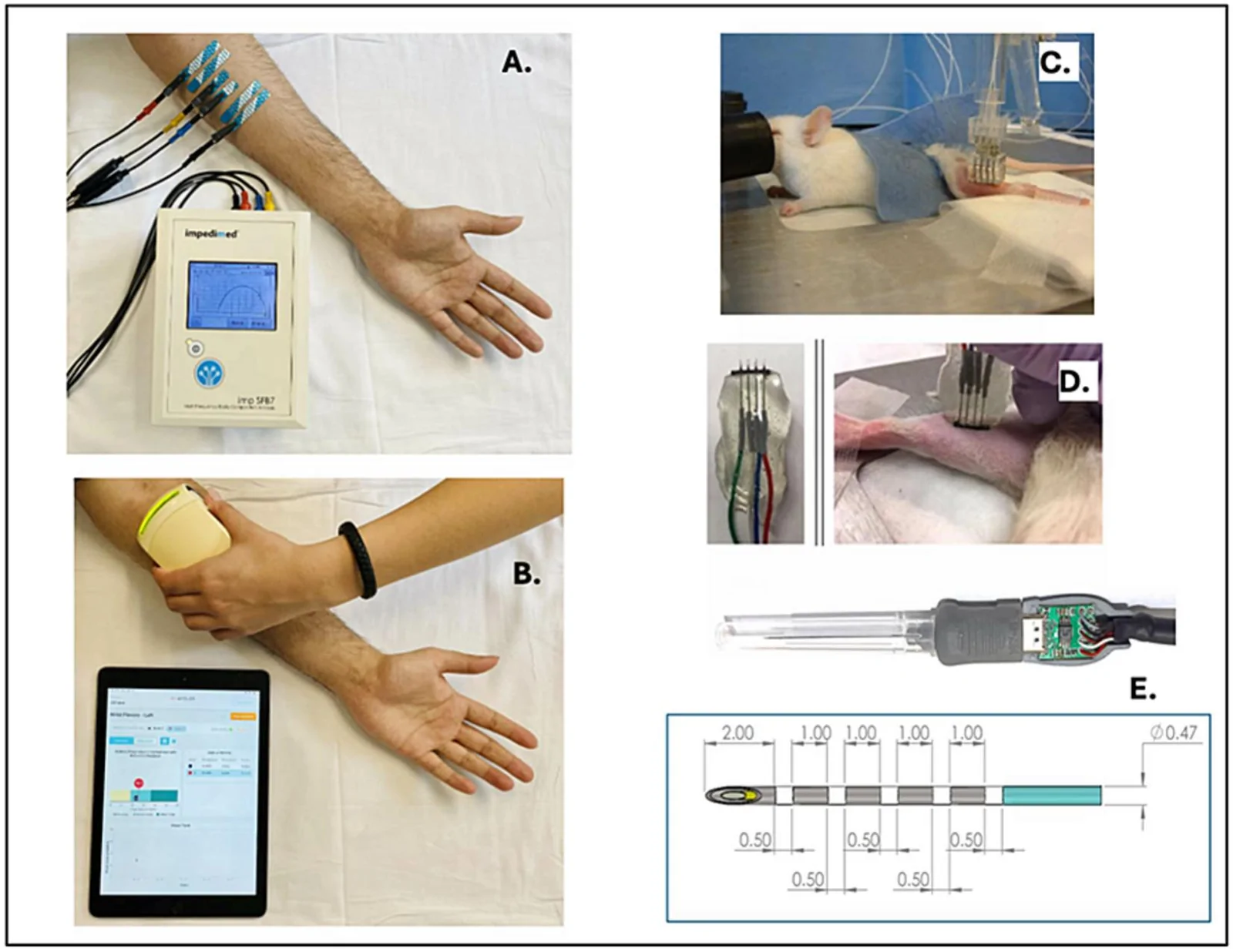
A sampling of different approaches for performing electrical impedance myography (EIM). A. Sensory nerve conduction electrodes used in conjunction the ImpSFB7 multifrequency bioimpedance device (Impedimed, Ltd), B. Handheld EIM system and iPad app (Myolex, Inc); disposable electrode array not visible; C. Surface electrode array being applied to the gastrocnemius of an anesthetized mouse with fur fully removed with depilatory agent; D. Multi-needle electrode array for use in rats, with fur clipped; E. Current version of impedance-EMG needle and schematic showing design that embeds 6 electrodes into the needle shaft, 2 for EMG and 4 for impedance.

EIM has been applied to a wide variety of muscular conditions mainly for the purpose of monitoring progression or therapeutic response, ranging from neurogenic diseases such as ALS (Shefner et al., 2018), spinal muscular atrophy (Cobb et al., 2024), radiculopathy (Spieker et al., 2013), and nerve injury (Li et al., 2017) to myopathic diseases, including various forms of muscular dystrophy (Leitner et al., 2020; Statland et al., 2016) and inflammatory myopathies (Roy et al., 2020). In addition to these “standard” neuromuscular diseases, EIM has been applied to other disorders which are not typically considered neuromuscular disease per se, including the evaluation of sarcopenia (Arnold et al., 2017) and disuse atrophy (Tarulli et al., 2009), as well as disuse changes associated with reduced gravitational loading (Semple et al., 2020). The specific alterations in EIM in disease states depend to some extent on the type and chronicity of the disorder present. One recent study demonstrated its potential as a primary neuromuscular diagnostic modality (Sonbas-Cobb et al., 2026).

While EIM has been studied in a wide group of disorders and by investigators and clinicians worldwide, this has been done mainly in the context of research (and to a lesser extent as part of outcome assessments in various clinical trials). The technology has not reached a wider audience to date. Part of the reason for this is that, at present, there is no approved (e.g., FDA- or EMA-cleared) EIM system. Many investigators perform data collection using off-the-shelf electrical impedance systems (developed for whole-body bioimpedance measurements or other non-medical applications). Nevertheless, commercial, dedicated devices have been developed for use in humans, both to measure surface impedances (Farid et al., 2022; Shefner et al., 2018) and to measure a combination of intramuscular electromyography-impedance myography signals (Rutkove et al., 2022). There remains no practical reason that EIM cannot continue to be advanced even in the absence of approved device.

### 7.2 Current applications of particular interest

Since the technique is entirely painless, has high reproducibility, and can be learned with minimal training, it has new applications beyond standard electrophysiological tests.

While a major focus of medical devices in neurology has been in diagnosis, today physicians are quite expert at this. Few disorders cannot be accurately identified through a combination of clinical, serological/genetic, histological, electrophysiological and radiological technologies and of course patience, effort, and financial support. With the advent of sophisticated clinical trials, and a plethora of new therapies, the focus of neuromuscular care has gradually shifted away from primary diagnosis to assessing therapeutic efficacy both in clinical trials and individual patient care.

EIM has the potential to provide rapid, non-effort-dependent, quantitative assessment of muscle condition and health, and response to therapeutic intervention. Indeed, some of the most promising applications of surface EIM techniques lie in this area. This has been especially true for muscular dystrophies (Leitner et al., 2020; Rutkove et al., 2017) and ALS (Shefner et al., 2018), where studies have shown that by utilizing EIM, the sample size to complete a clinical trial may be reduced. Importantly, EIM outputs correlate well with other accepted measures in these conditions (such as measures of muscle strength), supporting its validity.

Using impedance technology in the home is exceedingly simple. In fact, a commercial, at-home EIM-based measuring device, the *Skulpt Chisel®,* was marketed for several years. This system was able to capture data from several electrode arrays simultaneously at multiple frequencies and provide fitness enthusiasts with measures of muscle health and localized fat (McLester et al., 2018). The device was not formally registered as a medical product since it made no medical claims. The ability to measure oneself, or for a caregiver to measure a patient, is extremely valuable way of home monitoring which could be useful in clinical trials; more frequent measurements may allow subtler treatment effects to be identified by improving the signal-to-noise ratio (Rutkove et al., 2020). It could also enable remote data collection in patients receiving telehealth as part of their regular care. Currently, few such tools are available. A device that enables people to accurately measure their own muscles´ health themselves could add considerable value in the care of neuromuscular conditions.

Combining a measurement of active myofiber properties (provided by standard EMG) and passive muscle properties (provided by EIM) simultaneously would allow a muscle to be evaluated more completely than is currently possible. A prototype impedance-EMG needle has already been developed (Rutkove et al., 2022). If the technology can be commercialized, it would allow better muscle assessment of standard neuromuscular conditions and provide the opportunity to expand into conditions not classically considered neuromuscular per se, such as sarcopenia, cachexia, and disuse atrophy, in which the EMG signal is usually relatively normal. The technology remains in development, but the idea of embedding 6 electrodes (4 for impedance and 2 for EMG) in a single needle has been shown to be feasible and likely can be mass manufactured at acceptable cost. The two data sets could be acquired simultaneously or in quick succession during a single needle insertion.

AI and machine learning may be particularly well-suited for the interpretation of impedance data which are notoriously complex. EIM in its most simple formulation does not require tracking over seconds or minutes, as is the case with EMG, but there is a plethora of data. These include several basic impedance parameters (typically resistance, reactance, and phase angle) at dozens or even hundreds of applied frequencies, several derived parameters, and alternative electrode placements and orientations. This leads to complexity which is why published impedance work to date has focused on relatively simple outputs (e.g, the single frequency 50 kHz phase angle parameter). Much more, however, could be gleaned from the entire dataset than is currently achieved. AI and machine learning approaches seem promising to analyse this complex data set both to assist with classification questions (e.g., identifying a diagnosis) (Pandeya et al., 2022)and with tracking severity and progression (e.g., regression/prediction models) (Cobb et al., 2024). This entire area has been barely studied and in conjunction with the EMG (Pandeya et al., 2023) could offer even greater rewards.

Pediatric applications are of major interest. As noted earlier, a major advantage over standard NCS and EMG is that surface EIM is entirely painless and requires no active participation on the part of the patient; in fact, only a brief period of sitting still is required before the data is obtained. Moreover, unlike imaging modalities, it provides quantitative values that do not require expert review or complex algorithms to extract from a complex image. Thus, EIM data can provide insight into muscle health and insights into whether a therapy is working, either in a clinical trial or as part of individual patient care. Indeed, EIM has been studied in a variety of pediatric disorders including spinal muscular atrophy, Duchenne muscular dystrophy, and other hereditary myopathies. One other potential proposed use would be to help with an initial determination of whether a “floppy baby” being evaluated for the first time has central or peripheral hypotonia.

### 7.3 Future state

The application of electrical impedance techniques in the evaluation of neuromuscular disease is still in early stages. It remains unfamiliar to most electrophysiologists and is not taught in medical schools or training programs. Thus, how and when it will be more broadly incorporated into research or clinical practice remains uncertain. Several possibilities include:

Clinical therapeutic trials have become efficient by focusing on subsets of patients with more aggressive disorders to detect a treatment effect more readily, as was the case for the ALS drug edaravone (Abe et al., 2017) and in many others in which treatment groups are disproportionately represented to increase the likelihood that any given patient will be on the active drug. In parallel with these developments, home measurements in addition to in-person, in-clinic visits have become of interest (Daly et al., 2024). As described above, more frequent measurement improves signal, and thus a more accurate assessment of disease progression can be obtained for any individual patient (Rutkove et al., 2020). Unlike standard clinical neurophysiological outcomes (including even simple ones like CMAP), EIM makes it possible for data to be collected at home by patients or their caregivers on a very frequent basis (e.g., weekly or even daily). By measuring more frequently it becomes possible to detect a more modest drug effect and require fewer participants in a clinical trial. We could envision a time when neuromuscular clinical trials have both in-clinic assessments, requiring professional, trained personnel to perform assessments, and an at-home component in which a simple-to-use EIM device provides frequent, caregiver-obtained data directly and wirelessly to a central server.

As neuromuscular physicians expand the range of disorders that they treat and as pharmaceutical companies seek new markets, such as sarcopenia and cachexia, EIM may move to the forefront of assessment, either in clinical therapeutic trials or as part of standard care, to assess efficacy in an individual person, helping to direct medication or dosage choice. With an aging population, the importance of understanding and potentially ameliorating sarcopenia is of growing importance (Rolland et al., 2008). Similarly, there is increasing evidence that treating cachexia itself can help improve cancer survival (Mondello et al., 2014). Another area of growing importance is in GLP1-agonist and related weight-loss drugs. Whereas these drugs may be very valuable in losing fat, since people are simply eating less, they also tend to lose muscle (Prado et al., 2024). Use of EIM for regular assessment of muscle health in these common situations could become important. Finally, as described above, EIM may be useful in the broader fitness market.

The potential for impedance EMG is large. It could play a greater role in individual neuromuscular diagnosis, providing insights simultaneously into the passive and active electrical properties of the muscles. Moreover, like surface techniques, it could provide insight into therapeutic efficacy. Impedance EMG could be especially useful in situations where the muscles are deep, where there is considerable subcutaneous fat, or in which only certain specific muscles are affected, e.g. in focal nerve injury. It could also be used in clinical assessment of people in whom the focus is on assessment of non-myofiber aspects of the disease, such as connective tissue or fat deposition within the muscle.

Perhaps most futuristic is the potential for EIM to find use in assessing astronauts' health. Studies have been done in rodent models of space flight here on Earth as well as aboard the International Space Station (Semple et al., 2020; Sung et al., 2013). As people venture beyond low Earth orbit, to the Moon and then to Mars, it becomes important to consider regular evaluation of muscle condition, both to ensure the ability to perform regular tasks and to maintain broader health. Exercise and other countermeasures (electrical muscle stimulation and medications/supplements) are being studied as well to help maintain muscle and bone health during extended periods of reduced gravity. Having a convenient, lightweight tool to monitor muscle condition with simple readouts could be an important adjunct to ensure a successful mission to a distant goal and a safe return to Earth.

A final important aspect of EIM is in the assessment of muscle contraction, beyond that offered by EMG. Immediate impedance changes during isometric contraction are relatively small but become much larger in contractions in which the muscle and joint are allowed to move (e.g., isokinetic or isotonic contraction), as the muscle changes shape. As improved measurement protocols and techniques are developed, there is the potential for considerable advance in this area with a wide variety of applications, including muscle kinesiology, diagnosis and therapeutic monitoring, fitness, and rehabilitation (Xu et al., 2024).

## 8 Microneurography for studying the somatosensory system

Microneurography is an invasive technique for recording from peripheral nerve cells in awake humans developed in the Department of Clinical Neurophysiology in Uppsala in the mid-sixties (Hagbarth and Vallbo, 1967; Vallbo, 2018; Vallbo et al., 2004). It allows recordings from cutaneous afferents, muscle afferents, or sympathetic efferents. In this section we will focus on recordings from single nerve cells of the peripheral somatosensory system. Microneurography recording from the autonomic nervous system is presented elsewhere (Carter, 2019; Macefield, 2021).

During microneurography a thin tungsten electrode (diameter typically 200 μm with an uninsulated tip of 5 μm) is inserted through the skin into a peripheral nerve in a limb or in the face. Once inside the nerve the electrode is manually moved in fine steps until single afferent activity can be recorded. This has often been a tedious process, taking 2–3 h, as the experimenter relied on anatomical landmarks and electrical surface stimulation to localize the nerve. Now, with ultrasound for nerve visualization, finding the nerve is considerably easier and takes around 30 min, making microneurography suitable for more patients, though a session may still take up to three hours. The low yield, of typically 3–6 afferents per experimental session, is the main reason why microneurography is not suitable for routine diagnostic purposes. However, a variant of microneurography involving multiunit recordings of unmyelinated (C)-fibers and latency tracking of electrical stimulation has a higher yield (Serra et al., 1999) and is used in select patients with a suspicion of small-fiber neuropathy in a few laboratories in the world (Ochoa et al., 2005; Serra et al., 2014). This technique cannot record from large diameter fibers, however, because of their short latency responses to electrical stimulation.

Patients with mainly positive symptoms (i.e. pain or paraesthesia), in whom routine clinical neurophysiology tests are normal, may benefit from microneurographic recording from the sensory afferents which may be responsible for generating abnormal sensations. In the future, microneurography may be more widely used in this population, especially if the time for recordings can be shortened. This could be achieved by improved resolution of the ultrasound images which would make it easier to identify and impale the nerve. The experimental yield of axons recorded might be increased by using multi-site recording electrodes, as used for cortical recordings of patients with epilepsy (Chung et al., 2022). Multi-site electrodes can sample 100 cells at the same time, which would then be representative of the state of the nerve and hence be useful for diagnostics and a break-through for the microneurography technique.

Human nerve peripheral somatosensory nerve cells together with their cutaneous end organs are often referred to as “units”. The units are classified by their conduction velocity; rapidly conducting myelinated (A) or slowly conducting unmyelinated (C) unit types. Almost all myelinated units recorded with the microneurography technique belong to the thickly myelinated Aβ-class with conduction velocities >30 m/s. Units belonging to the thinly myelinated Aδ-class with a conduction velocity ∼ 10 m/s are rarely encountered in human microneurography (Nagi et al., 2019). The reason for this is unknown but could be due to technical difficulties in recording from thinly myelinated axons. Unmyelinated C-units with a conduction velocity of ∼1 m/s are often encountered. Units are further divided by their preferred stimulus into mechanoreceptors or thermoreceptors, and into low- or high-threshold. The high-threshold units are often referred to as nociceptors implying a role in pain processing and perception. The Aβ-mechanoreceptors are further subclassified into slowly or rapidly adapting types. One problem when recording from patients with a peripheral neuropathy is that it may be more difficult to classify the afferent into the same categories as for a healthy nerve.

In the future, microneurography could adopt methods for unbiased classification of nerve types, inspired by transcriptomic approaches developed for classification of peripheral nerve cells (Usoskin et al., 2015). If all units are stimulated, in microneurography, using the same standardized stimulation protocol, it should be possible to apply tools for dimensionality reduction and visualize those results in two-dimensional uniform manifold approximation and projection (UMAP) plots. Dimensionality reduction is a technique that helps reduce many-dimensional data into just two dimensions. UMAP plots are a human-friendly way to visualize the results where units with similar response properties form distinct clusters. The standardized stimulation protocol needs to cover a range of different types of stimuli, i.e. mechanical, electrical, thermal, and chemical stimuli (such as capsaicin, menthol, and mustard oil) so that the different unit types can be disentangled. We would expect the UMAP plots to look similar across healthy subjects, but we may start to see deviations in pathological states. UMAP data representation may allow the detection of patterns of peripheral unit pathology specific for different diseases and follow up of the effects of pharmacological or other types of treatments.

Another future direction is to combine microneurography with single-cell RNA-sequencing of nerve cells taken from dorsal root or trigeminal ganglia from deceased human donors. Single-cell RNA-sequencing of dorsal root ganglia from donors without any known disease (death from accidents) has demonstrated that some human peripheral unit types unexpectedly express the mechanically sensitive PIEZO2 ion channels as well as the temperature sensitive TRPV1 and TRPM8 ion channels (Yu et al., 2024). The functional significance of these expressions was tested in microneurography and consistently showed a previously unknown polymodality in some unit types (Yu et al., 2024). For example, a subtype of the so-called ultrafast nociceptors, previously thought to be pure mechanoreceptors, expresses PIEZO2 and TRPM8 and respond to not only mechanical but also cooling stimuli (Yu et al., 2024) (Fig. 10). Another example is unmyelinated low-threshold mechanoreceptors that express PIEZO2 and TRPV1 and respond not only to mechanical but also to warming stimuli. Curiously, the unmyelinated low-threshold mechanoreceptors do not express TRPM8, yet they show robust cooling responses in microneurography (Fig. 11). The cooling response of low-threshold unmyelinated mechanoreceptors thus has to be mediated by a non-TRPM8 mechanism that remains to be identified. A next step will be to perform similar type of molecularly informed microneurography in patients with diseases affecting the somatosensory system. Single-cell RNA-sequencing could be done in deceased donors who suffered from conditions such as fibromyalgia, migraine, or diabetic neuropathy. The functional significance of any disease-specific transcriptome abnormality could then be evaluated in microneurography recordings in living patients with the same disease as the deceased donor suffered from. Such a combination of genetical and functional data may provide novel information for understanding disease mechanisms at functional and cellular levels – a cornerstone of precision medicine.

**Fig. 10:**
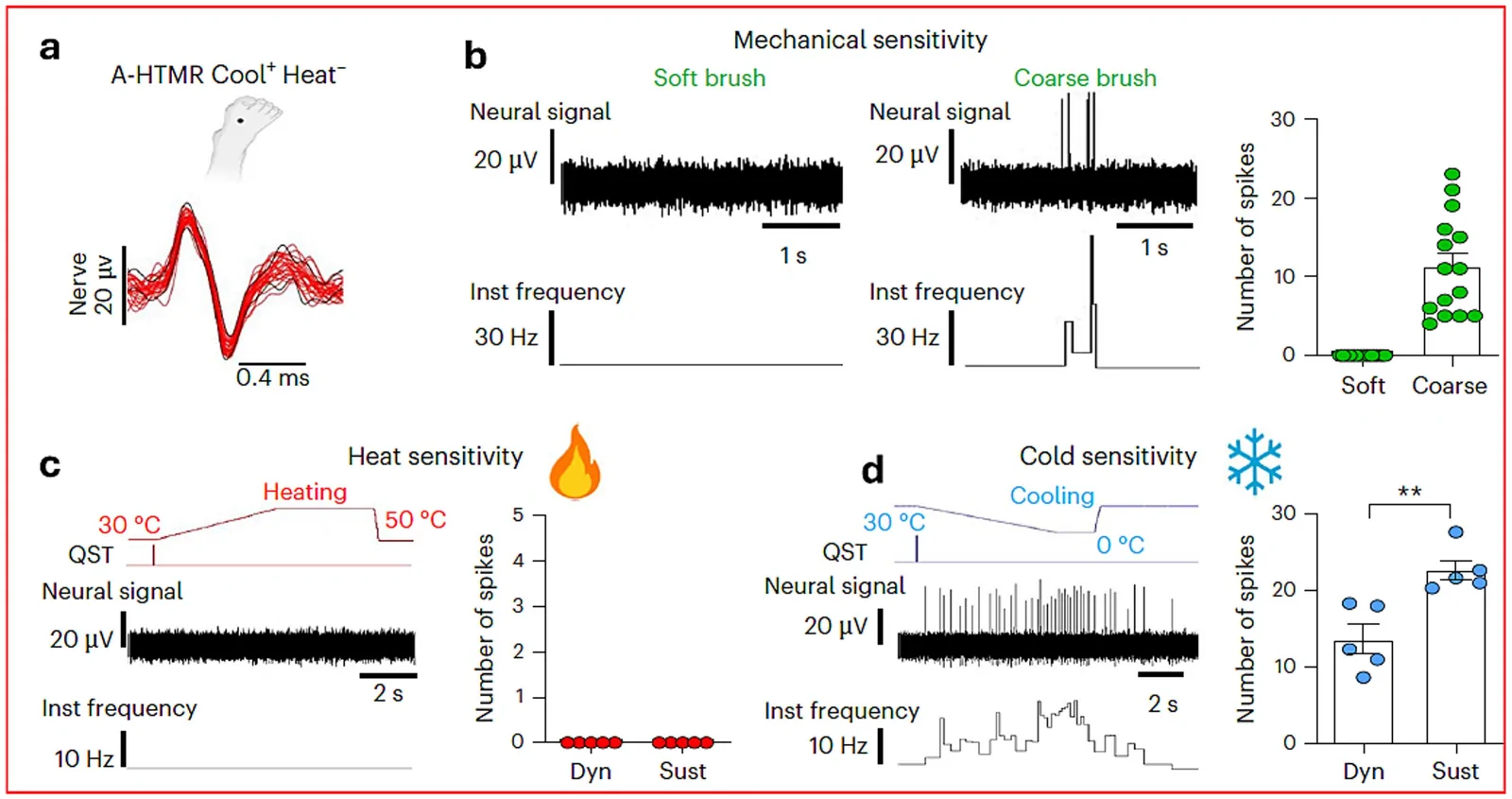
Transcriptomics-informed single-unit microneurography recordings of human A-fiber high-threshold mechanoreceptors (A-HTMRs). a, Superimposed spike activity of an A-HTMR Cool+ Heat− unit in response to repeated sensory stimulation of the receptive field. The receptive field location is indicated by the blue dot. b–d, Individual and mean (+/− s.e.m.) responses of A-HTMR Cool+ Heat− units to brushing (b), heating (c) and cooling (d). Transcriptomic analyses indicate that A-HTMR Cool+ Heat− neurons lack expression of the mechanosensitive ion channel PIEZO2. Consistently, these units showed no response to soft brushing, but all responded to coarse brushing (*n* = 5 units, each tested in triplicate, all trials shown) suggesting that high-threshold mechanical responses may be mediated by mechanisms independent of PIEZO2. Similarly, transcriptomic data indicate an absence of the heat-sensitive ion channel TRPV1, and no responses to heating were observed. In contrast, transcriptomic data indicate expression of the cool-sensitive ion channel TRPM8, and all units responded to cooling during both the dynamic (4 °C per s) and sustained phases. Each data point for heating and cooling represents the mean of triplicate responses from an individual unit (*n* = 5 units). An increased response was observed during sustained cooling compared to dynamic cooling (two tailed paired *t*-test: *t*(4) = 4.889, *P* = 0.0081). Adapted from Yu et al., 2024. (For interpretation of the references to colour in this figure legend, the reader is referred to the web version of this article.)

**Fig. 11:**
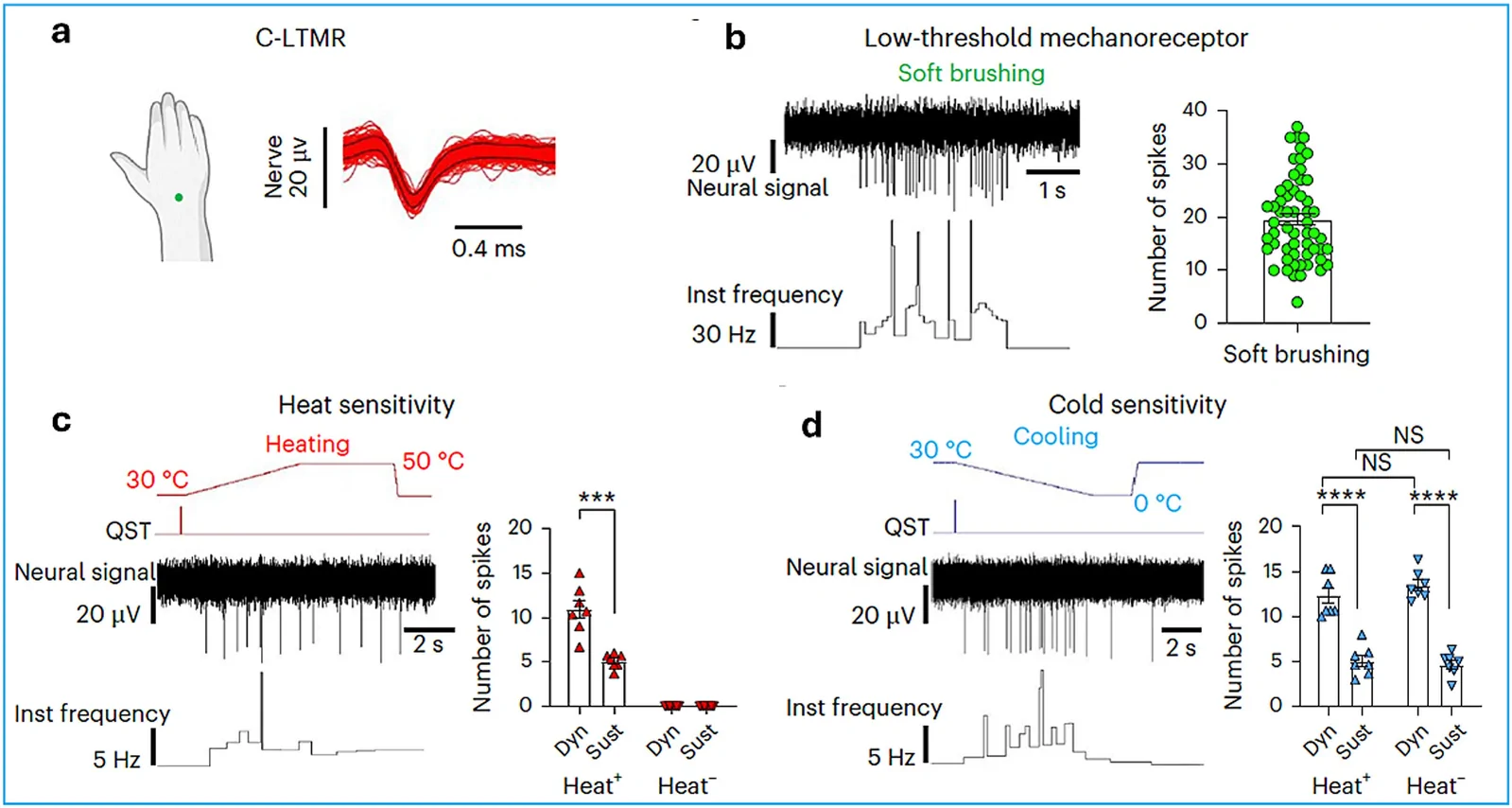
Transcriptomics-informed single-unit microneurography recordings of human C-fiber low-threshold mechanoreceptors (C-LTMRs). a, Superimposed spike activity of a C-LTMR in response to repeated sensory stimulation of the receptive field (marked by the green dot). b-d, Individual and mean (+/− s.e.m.) responses of C-LTMRs to soft brushing (b), heating (c) and cooling (d). Transcriptomic analyses indicate that C-LTMRs express the mechanically sensitive PIEZO2 ion channel and the units responded robustly to soft brushing (*n* = 17 units, 57 trials, all displayed). Transcriptomic data further indicate expression of the heat-sensitive ion channel TRPV1. Heating was tested in 14C-LTMRs, of which seven responded. Each data point represents the mean of triplicate responses from one unit. Heat-responsive C-LTMRs showed greater responses during dynamic than sustained heating (*n* = 7 units; two-tailed paired *t*-test: t(6) = 6.591, *P* = 0.0006). In contrast, transcriptomic data suggest that C-LTMRs do not express the cool sensitive ion-channel TRPM8. Nevertheless, all 14 units responded to cooling suggestive of a non-TRPM8 mediated cooling response. Each data point represents the mean of triplicate responses from one unit, with increased responses during dynamic cooling compared to sustained cooling (two-way ANOVA: *F*1,24 = 144.6, *P* < 0.0001; Tukey's test: *P* < 0.0001). No significant differences (*P* > 0.05) were observed in cooling (d) between heat-responsive and non-heat responsive C-LTMRs (two-way ANOVA: *F*1,24 = 0.2860, *P* = 0.5977). Adapted from Yu et al., 2024. (For interpretation of the references to colour in this figure legend, the reader is referred to the web version of this article.)

Traditionally in microneurography, single units have been stimulated using well-defined stimuli, with a move from handheld devices to robotic devices for high-precision tactile (Jarocka et al., 2021; Loken et al., 2009) or proprioceptive stimulation (Jones et al., 2001) and Peltier elements or laser for precise thermal stimulation (Dusch et al., 2016; Weinkauf et al., 2015). Units may also be characterized by their responses to different patterns of electrical stimulation (Schmidt et al., 1995; Watkins et al., 2017) or using various types of chemical compounds such as capsaicin, menthol, or BAM 8–22 (Fiebig et al., 2023).

Though more standardized and ‘pure’, these stimuli are vastly different from the complex touch experiences we encounter in daily life. An alternative strategy is to use naturalistic stimuli such as human-to-human skin stimulation where the experimenter strokes, squeezes, or presses the skin of the subject using his/her hand (Xu et al., 2022). To correlate neural responses to naturalistic touch stimuli requires the precise measurement of human skin-to-skin contact simultaneously with the microneurography recording. Such interference-free measurements of skin deformation are technically challenging. Recently this was managed using a 3D visual tracking system employing a depth camera to measure the skin deformation caused by the experimenter's hand or fingers touching the subject's skin (Xu et al., 2022). The skin-to-skin interaction can then be decomposed into time-series of high-resolution contact attributes including contact area, indentation depth, absolute velocity, and three orthogonal velocity components. The contract attributes can then be correlated with the neural responses from simultaneous microneurography recordings. Using naturalistic stimuli, instead of highly controlled experimental stimuli, is particularly relevant in social touch research and may allow the identification of the peripheral neural underpinning of human-to-human touch communication (McIntyre et al., 2022). This may be relevant for the understanding mechanisms of touch avoidance seen in conditions such as autism spectrum disorders (Croy et al., 2016).

In conclusion, the low yield of microneurography may be dramatically improved with the development of multi-site electrodes. Difficulties in categorising recordings from units in patients, because afferent responses from a damaged nerve may differ from that in a healthy nerve, may be mitigated by using a standardized stimulation protocol followed by unbiased classification based on dimensionality reduction and UMAP clustering analyses. Recent transcriptomic analyses of human dorsal root and trigeminal ganglia open up the possibility of studying functional consequences of molecular expression in healthy and pathological conditions. Finally, the emerging area of social neuroscience will benefit from using naturalistic human-to-human touch to identify the peripheral neural origin of emotions evoked by touch. Microneurographic single-unit recordings from peripheral nerves in awake humans has been used for 60 years. This section has highlighted some ways in which it may be developed over the years and decades to come.

## 9 Nerve excitability testing

NCS give information about the number of conducting nerve fibers and their conduction velocity which is typically used to localize the site of a pathology, determine whether the pathology is due to demyelination, axonal loss, neuromuscular junction disorder, or entrapment, and to monitor progression of diseases. However, traditional NCS are unable to give further insight into the dysfunction of the nerve membrane – which of course is central to impulse propagation.

Nerve excitability testing is a technique which is complementary to conventional NCS. It is non-invasive and an in vivo analogue of the current-, voltage- and patch- clamp techniques used in electrophysiology to probe membrane and ion channel function. Nerve (or axonal) excitability testing uses a very similar stimulating and recording arrangement to NCS. It is quick, easy to perform and is applicable to any accessible nerve. It is also extremely sensitive to membrane potential, ion channel properties and the integrity of the myelin sheath and its attachment to the axon (Garg et al., 2019; Garg et al., 2018; Kiernan and Bostock, 2000; Kiernan et al., 2005a; Nodera et al., 2004; Tomlinson et al., 2018).

### 9.1 Overview of nerve excitability testing techniques

There is a long history of nerve excitability testing, but the modern technique incorporating multiple excitability was born out of a Summer Course in ‘Clinical Axonal Electrophysiology’ in Trondheim Norway in 1999. The ‘*TROND*’ protocol standardized these tests, and the participants from that course provided the first normative data for axonal excitability of motor axons, followed shortly thereafter by characterization of sensory axons in the median nerve of healthy subjects (Kiernan et al., 2000; Kiernan et al., 2001).

Modern excitability measurements are essentially a stimulus-response paradigm which uses automated threshold-tracking techniques to test the excitability of axons at the target level (Fig. 12A). In this context ‘threshold-tracking’ refers to the automatic adjustment of the strength of a stimulus until a specific nerve response is observed, similarly to a thermostat maintaining room temperature by adjusting heating or cooling.

**Fig. 12:**
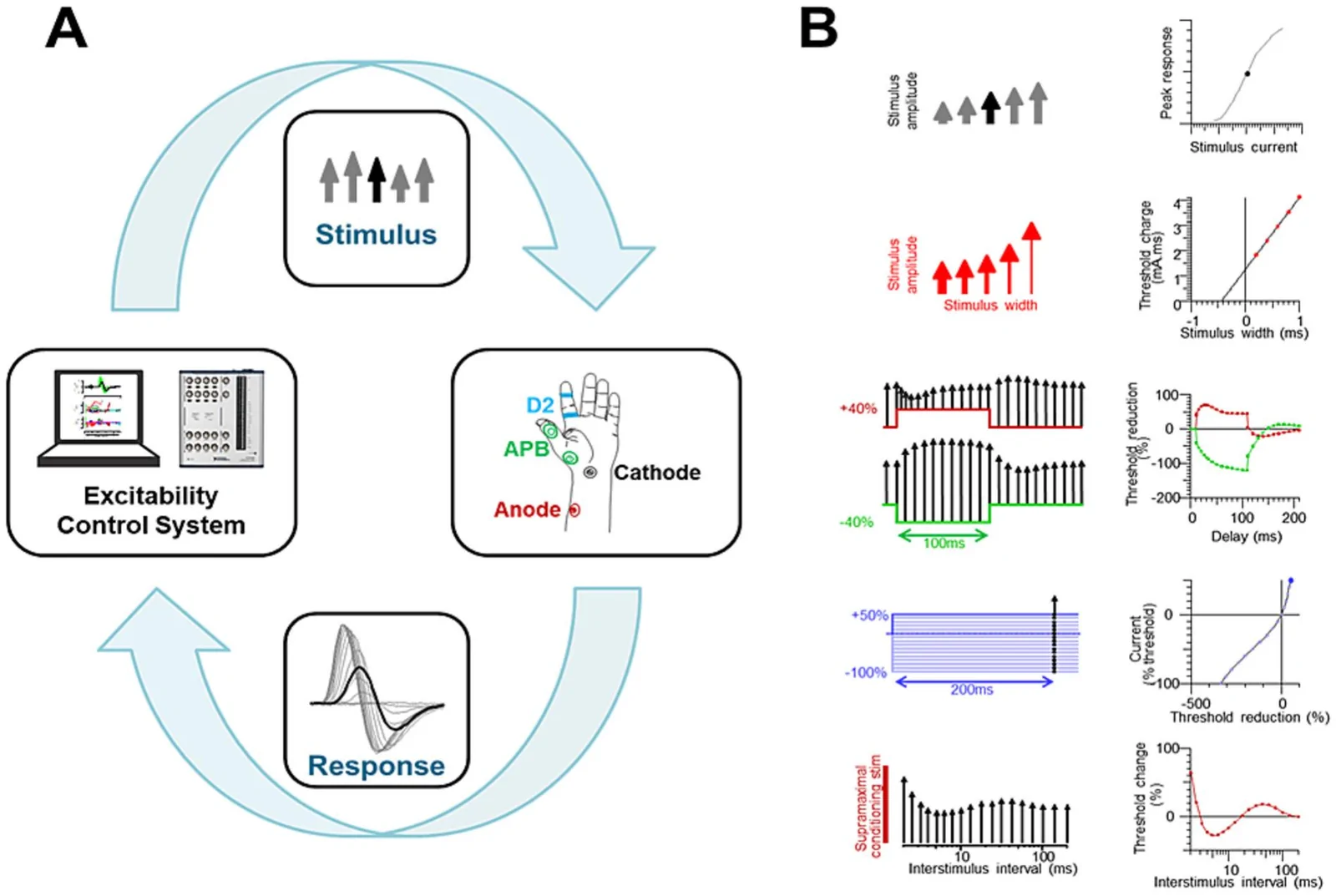
A. Overview of the setup for axonal excitability measurements. The excitability is measured by tracking the stimulus intensity (‘threshold’) required to elicit a target response (usually 40% of the maximal motor and sensory action potential). B. Overview of the TROND multiple excitability measures protocol. *Stimulus/response relationship*; response to stimuli of increasing strength from subliminal to maximal. Target response is a fixed fraction of the maximal response (shown by the filled circle in the top plot). The *strength-duration properties* examine the relationship between stimulus width and stimulus strength required to elicit the target response. *Threshold electrotonus; axons'* excitability before, during and after long-lasting subthreshold conditioning stimuli to study axons' accommodative properties. *Current-threshold relationship;* excitability after a 200 ms conditioning stimulus which varies in amplitude from 50% to −100% of the control amplitude. *Recovery cycle;* recovery of excitability after a supramaximal stimulus, with a stereotypical pattern of refractoriness, superexcitability and then late subexcitability.

The stimulus strength (termed the ‘threshold’) is a measure of axonal excitability and is tested under a variety of conditions, which are described below.

### 9.2 Stimulating and recording setup

Nerve excitability studies typically use low-impedance, self-adhesive non-polarizable Ag/AgCl ECG electrodes for stimulation. A monopolar montage is used to assess the excitability of the nerve directly under the stimulating cathode, with a remote anode. In contrast to NCS, stimulation is usually provided by an isolated constant-current stimulator that can deliver a continuous arbitrary-waveform current which can be controlled in real time.

The recording electrode setup is the same as with conventional NCS with the active electrode (E1) over the muscle or nerve segment, and the reference electrode (E2) located away from active muscle for motor, or distal to the active electrode for sensory studies (Fig. 12A). Responses are amplified and filtered by an isolated amplifier and then line-frequency (50/60 Hz) noise is removed before digitization by a data acquisition system. Most excitability recordings to date have been performed using the ‘Qtrac’ excitability software, though future possibilities for more widespread adoption of the techniques are discussed later.

### 9.3 Multiple excitability measures: The TROND protocol

The standard *TROND* excitability protocol provides a comprehensive assessment of axonal excitability in 10–15 min and consists of 5 main parts (Fig. 12B).

The ***stimulus-response curve*** is typically sigmoidal in nature and measures the peak response to increasing stimulus current intensities. It is also used to set the target response (typically 40% of the maximal response) for the subsequent tests and is used to optimize the tracking of threshold changes in the subsequent tests.

The ***strength-duration properties*** are measured by finding the stimulus current amplitude (termed threshold) required to elicit the target response to stimuli of different widths. When the stimulus charge (μC; threshold [mA] × width [ms]) is plotted against the stimulus width, the slope is the rheobasic current, and the magnitude of the x-axis intercept is the strength-duration time constant.

The *strength-duration time constant* is affected by both persistent Na^+^ currents and passive membrane properties.

***Threshold electrotonus*** is measured by testing the threshold before, during and after long-lasting (100 ms) conditioning stimuli whose amplitudes are ±20, ±40% of the unconditioned control stimulus threshold.

When the excitability is plotted as a threshold reduction, *threshold electrotonus* strongly resembles the electrotonic changes in membrane potential when long-lasting currents are applied in current-clamp recordings.

The ***current-threshold relationship*** also assesses the effect of long subthreshold stimuli, but it does so by testing the threshold at the end of 200-ms current stimuli which varies in intensity from +50% (depolarizing) to −100% (hyperpolarizing) of the unconditioned control stimulus threshold.

When the current is plotted against the threshold-reduction, the resulting curve is analogous to the conventional current-voltage ‘I/V' plots of electrophysiology.

The ***recovery cycle*** is measured by testing the threshold following a supramaximal stimulus at 18 conditioning-test intervals which are approximately logarithmically spaced from 2 to 200 ms. Recovery of excitability following activation follows a typical pattern of absolute refractoriness, relative refractoriness, superexcitability and then late subexcitability.

There are overlapping mechanisms underlying the recovery cycle, and subsequent phases are affected by the resting state or earlier phases of excitability. However, refractoriness usually represents the inactivation of Na^+^ channels and the activity of fast K^+^ channels. The period of superexcitability usually reflects a return of charge which was stored on the internode during the action potential (Barrett and Barrett, 1982). Subexcitability represents the continued activation of slow K+ channels that slowly close.

Changes to membrane potential, ion channel function or axonal structure will often manifest in stereotypical patterns of change in multiple excitability measures. As such, it is important to consider all elements of the TROND protocol together when interpreting changes in excitability.

### 9.4 Interpretation of changes and the ‘Bostock’ model of axonal excitability **(**Fig. 13**)**

The principal determinants of nerve excitability measured using these threshold-tracking techniques are: the voltage-gated ion channels, fast and persistent Na^+^ channels, slow and fast K^+^ channels, HCN channels; ohmic leak channels; the electrogenic Na^+^/K^+^ pump and the Barrett-Barrett pathways through and under the paranodal myelin sheath (Fig. 13A; Barrett and Barrett, 1982; Bostock et al., 1991; Bostock and Rothwell, 1997).

**Fig. 13:**
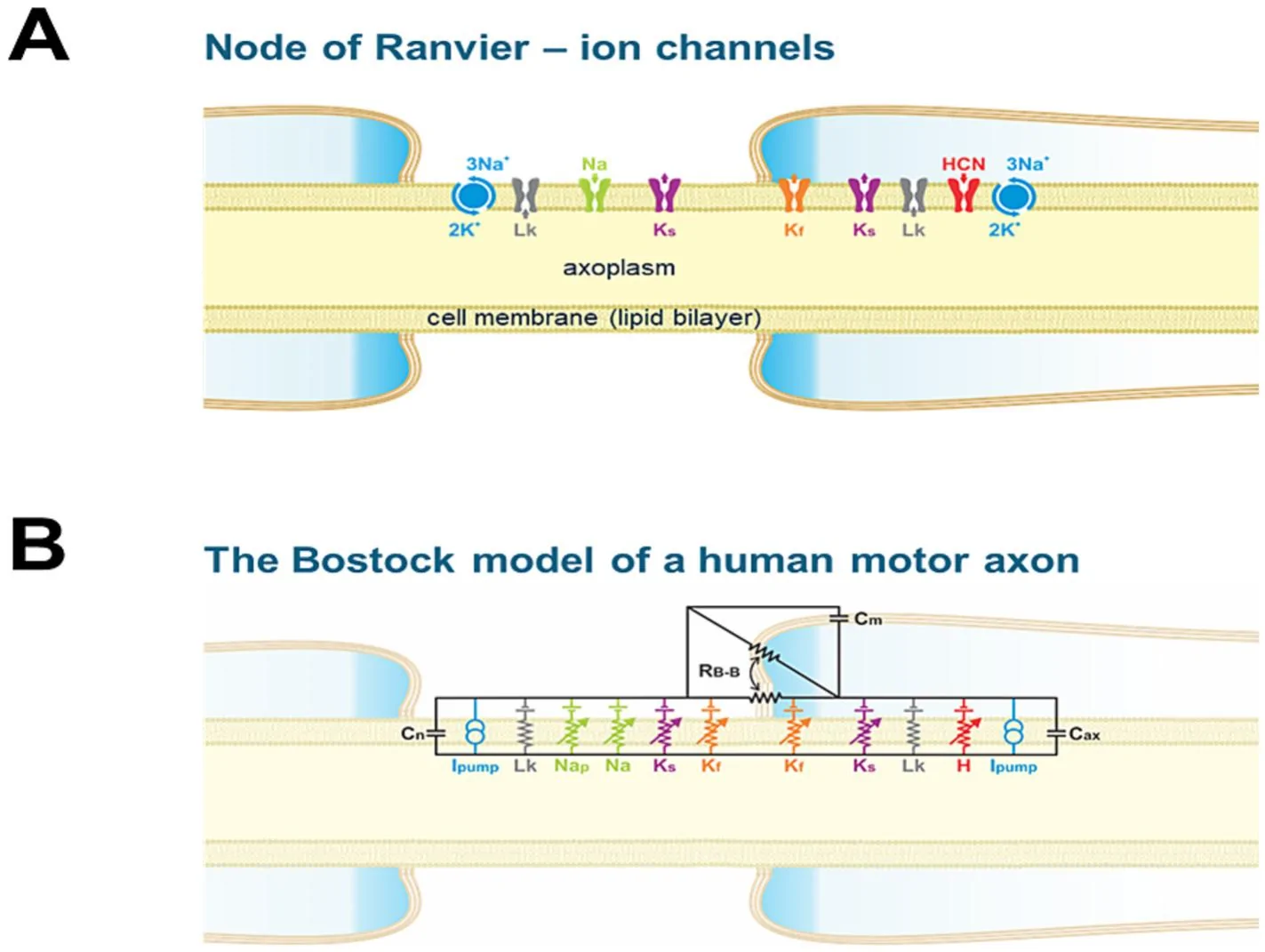
A. The principal determinants of axonal excitability as measured using threshold-tracking techniques. The electrogenic Na^+^/K^+^ pump, leak channels, fast and persistent Na^+^ channels, slow and fast K^+^ channels, and Hyperpolarization-activated Cyclic Nucleotide-gated (HCN) channels. B. The Bostock model of a human motor axon, which mathematically models the excitability of a human axon in terms of an equivalent electrical circuit.

Many of the changes in axonal excitability can be interpreted with a good understanding of the effects of membrane potential and the role of rectifying channels (slow and fast K^+^ and HCN) on axonal excitability (Baker et al., 1987; Kiernan and Bostock, 2000).

To support the interpretation of changes in axonal excitability, a simple model of a human motor axon (the ‘Bostock’ model) has been developed and it has subsequently been adapted to study the excitability of human sensory axons, and mouse axons (Fig. 13B; Bostock et al., 1991; Howells et al., 2012; Kiernan et al., 2005a; Makker et al., 2018).

### 9.5 Utility of nerve excitability testing

Nerve excitability testing is very sensitive to changes in resting membrane potential, ion channel function and axonal integrity at the site of testing. Changes in the excitability of peripheral axons may even be observed in conditions with a more proximal site of onset (see the discussion of benign familial neonatal epilepsy and stroke below), and differences in axonal excitability may predispose an individual to symptoms.

Nerve excitability testing has been used to gain further insight into the underlying mechanisms of neuropathic disease, and to assess therapeutic interventions and disease progress. Below are some examples highlighting the utility of nerve excitability testing organized according to the underlying mechanisms.

### 9.6 Resting membrane potential and ischaemia

Nerve excitability testing is very sensitive to changes in resting membrane potential. Depolarization of resting membrane potential with weak DC currents changes most excitability parameters (see Fig. 2 in Kiernan and Bostock (2000)), but apart from the expected decrease in threshold and rheobase, the most conspicuous changes are the increase in accommodation during threshold electrotonus and the marked shift of the recovery cycle with greater refractoriness, reduced superexcitability and increased late subexcitability. Conversely, hyperpolarization of resting membrane potential increases threshold and rheobase, decreases accommodation and shifts the recovery cycle toward reduced refractoriness, increased superexcitability and decreased late subexcitability.

Ischaemia and release from ischaemia produce patterns of excitability very similar to those observed with pure depolarization and hyperpolarization, respectively. However, the changes in late subexcitability due to pure polarization of resting membrane potential differs from that seen with ischaemic depolarization and post-ischaemia, which is likely due to the expected changes in extracellular K^+^ levels during and post ischaemia.

***Na***^***+***^ ***channels:*** Unsurprisingly, tetrodotoxin poisoning (from ingestion of puffer fish) has a profound effect on axonal excitability (Kiernan et al., 2005a). In addition to the expected changes of increased stimulus threshold, reduced compound responses and delayed latencies, axonal excitability revealed abnormalities in all elements of the *TROND* protocol. Tetrodotoxin is a potent blocker of Na^+^ channels, and all changes in motor axon excitability observed with the drug were adequately modelled by a halving of the maximum Na^+^ permeability. In a cohort of patients with generalised epilepsy with febrile seizures, axonal excitability in peripheral nerves revealed a pattern of excitability similar to that due to tetrodotoxin block, albeit to a lesser extent (Kiernan et al., 2005b). The authors concluded that the established mutation in the Na^+^ channel subunit of these patients resulted in a reduction in the number of functioning Na^+^ channels rather than a change in gating. Nerve excitability testing has also been demonstrated to be an effective pharmacodynamic marker of voltage-gated Na + channel blockers such as mexiletine, lacosamide and lidocaine (Moldovan et al., 2014; Ruijs et al., 2022). Acute administration of oxaliplatin shows changes consistent with Na^+^ channel inactivation and reduction of fast K^+^ conductances (Heide et al., 2018; Makker et al., 2020; Park et al., 2011).

***Fast K***^***+***^ ***channels:*** The axonal excitability of patients with episodic ataxia type 1 (mutation in the KCNA1 gene encoding the fast K^+^ channels) resembles a partial block of fast K^+^ channels by 4-aminopyridine (Baker et al., 1987; Tomlinson et al., 2010b). However, patients with acquired neuromyotonia due to autoantibodies to voltage-gated potassium channels have relatively normal axonal excitability. This may reflect the relative difficulty for autoantibodies to gain access to fast K^+^ channels located at the juxtaparanode, which are protected by the blood-nerve barrier, as opposed to those located at the relatively exposed motor terminals.

***Slow K***^***+***^ ***channels:*** Mutations in the KCNQ2 gene of patients with benign familial neonatal epilepsy result in a loss of function of the K_v_7.2 subunit in slow K^+^ channels, which is readily seen in depolarizing threshold electrotonus with a reduction in the accommodation to depolarization and in the recovery cycle with reduced refractoriness and increased superexcitability (Tomlinson et al., 2012). It is worth noting here, that this is one of many examples of the utility of testing the excitability of peripheral axons in CNS disorders. It works here because the affected ion channels (K_v_7.2) are expressed in both the central and peripheral nervous systems, but some other CNS insults such as stroke appear to affect the excitability of downstream axons, which is discussed next.

***HCN channels:*** Unlike most other excitability parameters which vary little between subjects, accommodation to hyperpolarization appears to be characteristic of an individual (Tomlinson et al., 2010a). Indeed, modulation of the voltage dependence of HCN channels adequately explains most of the variability between healthy subjects in both motor and sensory axons (Howells et al., 2012). In patients with unilateral hemiparesis due to stroke, a reduction in accommodation to hyperpolarization in motor (but not sensory) axons was observed in the affected limb, suggestive of a downstream modulation of the hyperpolarization-activated current *I*_h_ (Jankelowitz et al., 2007).

An increase in *I*_h_ has been observed in motor axons of patients with benign cramp fasciculation and idiopathic restless legs syndromes suggesting a role for HCN channels in the pathophysiology (Czesnik et al., 2019; Czesnik et al., 2015).

### 9.7 Axonal structure

The nerve excitability of chronic inflammatory demyelinating polyradiculoneuropathy (CIDP) patients with antibodies to neurofascin-155 is markedly abnormal. An increase in the ‘Barrett-Barrett’ conductance between the node and internode adequately explained the abnormal excitability and the elevated thresholds for stimulation.

### 9.8 Future

Modern excitability studies were born out of research by Bostock and colleagues and have almost exclusively used the QtracW software and which was developed for in vivo human studies research (Bostock et al., 1998; Burke et al., 2001; Kiernan et al., 2000). The continued success of nerve excitability testing has led to its use in clinical trials, drug development, and by a limited number of groups as a clinical tool to complement NCS. Widespread use of nerve excitability testing in the clinical domain will depend on several factors.

Most of the nerve excitability studies to date have been performed with the Qtrac excitability software which is incredibly flexible but is geared toward research. Simplification of the presentation of nerve excitability techniques and results and minimising excitability jargon will greatly assist the appeal to non-specialists. The techniques and the results are no more complex than many other established and accepted techniques in medicine and are rooted in the fundamental electrophysiology that underpins our understanding of the nervous system. The DS5 constant-current stimulator (Digitimer Ltd) is still the only medically certified device that is capable of real-time control of continuous stimulus output and is essential for many parts of the TROND protocol, particularly the long-lasting conditioning stimuli of threshold electrotonus and the current-threshold relationship.

Most EMG machines should be able to deliver programmable, short rectangular current pulses, which would make implementation of the stimulus-response and strength-duration relationships straightforward (Tyberghein et al., 2023). It may even be possible to incorporate the recovery cycle into existing EMG hardware. However, what most EMG machines will not be able to do with existing hardware is to deliver long-lasting current stimuli (≥ 100 ms). The DS5 constant-current stimulator is still the only medically certified device that is capable of real-time control of continuous stimulus output and is essential for studying threshold electrotonus and the current-threshold relationship, which give insight into the function of slow and fast K^+^ and HCN channels. The fact that there is already an existing certified isolated constant-current stimulator on the market removes the biggest barrier to the incorporation of nerve excitability techniques into commercial EMG machines.

Axonal excitability testing is now well established, and although the mechanistic insights from many neuropathies have already been studied, it will continue to provide insight into dysfunction of the axonal membrane in disease. Most studies to date have used the changes in excitability of motor axons studied at the level of the wrist, as a marker of systemic change, but there are differences in the excitability of motor and sensory axons at different sites which need further exploration.

The ability to study both motor and sensory axons at the same site of stimulation allows us to examine neuropathies that have systemic changes, or those that are specific to sensory or motor axons. Similarly, changes in the axonal excitability of these large, myelinated axons may provide a surrogate for small fiber dysfunction in some conditions.

Differences in cohorts, sites of study, disease progression, response to therapeutic intervention and drug development mean that axonal excitability studies will remain valuable for some time to come.

Future advances in nerve excitability may come from combining existing and future data in a large publicly accessible database. Challenges for such a goal include: cost and reward for effort (e.g. data collection, quality control, storage and access management), mechanisms for sharing and various regulatory requirements regarding privacy, ethics and data sharing and protection. However, the benefits of sharing data far outweigh the challenges. A well-constructed database of normative and disease group data could provide an invaluable resource for research and training, help define cut-offs etc. for diagnostic purposes, and increase the accuracy of correlations with temperature, age, sex and other clinical variables.

One of the strengths of nerve excitability testing is that changes in the excitability results are often characteristic or stereotypical in nature and are strongly suggestive of a particular dysfunction of ion channel / membrane potential behaviour. However, it could be argued that interpreting excitability in this manner has a particular bias, and that an artificial intelligence model could provide a fresh (unbiased) approach to the interpretation of nerve excitability results. If excitability datasets are more widely shared, then the benefits of big data with artificial intelligence could then be more readily realised.

Nerve excitability data is often summarized using derived excitability variables, but there may be hidden associations within the underlying excitability waveforms, that could be revealed with artificial intelligence techniques. Statistical methods such as sensitivity analysis and principal components analysis of axonal excitability data from patients with amyotrophic lateral sclerosis have already been used to extract further insight (Lugg et al., 2023; Stikvoort Garcia et al., 2024; Stikvoort Garcia et al., 2023). Artificial intelligence could also be used at the signal processing stage to improve the signal to noise ratio.

In conclusion, the continued strength of nerve excitability techniques is due to the tight connection between human clinical studies and pre-clinical animal studies, and traditional electrophysiology. The ‘Bostock’ mathematical model is a simple yet effective tool to assist interpretation and for interpreting differences between study targets. Nerve excitability techniques are well suited for revealing underlying neuropathic mechanisms, assessing drug target engagement and monitoring of therapeutic efficacy or disease progression (Kovalchuk et al., 2018; Lin et al., 2024; Wainger et al., 2021). Efforts to make the technique more accessible and simplify the presentation will only enhance its utility. Data sharing and collaboration will further enhance the value of nerve excitability testing by enabling larger datasets for analysis, facilitating multi-centre studies, and accelerating the discovery of new diagnostic markers.

## 10 Muscle excitability testing

### 10.1 Introduction

Muscle excitability testing provides in vivo information about muscle fiber membrane properties such as membrane potential and ion channel function and is derived from well-established neurophysiological methods (Tankisi et al., 2024a). It is based on recording velocity recovery cycles of muscle fibers with one or more conditioning stimuli and variable interstimulus interval. Velocity recovery cycles were proposed for the first time by Bergmans (Bergmans, 1971) as an indirect way to measure the negative afterpotential (Bergman and Johns, 1971). Earlier reports on absolute membrane potentials in vivo in healthy humans and in disease (Beranek, 1964; Cunningham Jr. et al., 1971; McComas et al., 1968) had used intracellular recordings with glass micropipettes which was impracticable in routine diagnostics. Though several studies had recorded velocity recovery functions of muscle fibers in vivo, (Allen et al., 2008; Mihelin et al., 1991; Stålberg, 1966), these studies used single muscle fiber recordings, which needed special recording needles, a low noise environment and can be time consuming since solation of the same single muscle fiber for stimulation and recording can be very difficult in a 3D structure such as a muscle.

### 10.2 Muscle excitability testing using multi-fiber stimulation

This changed in 2009, with a semi-automated technique for recording velocity recovery cycles of multiple muscle fibers, later called ***“muscle excitability testing”***, (Z'Graggen and Bostock, 2009; Tankisi et al., 2024a) (Fig. 14). The multi-fiber stimulation approach is easier, faster to perform and requires less training and skill to perform. It does not require much cooperation from the patient either. In addition, advances in the QtracW software for recording and analysis have further simplified and accelerated examination. An average examination now takes 10 to 15 min. The current software version includes a protocol recording multi-fiber velocity recovery cycles with one, two and five conditioning stimuli, a frequency ramp protocol to explore the effect of more extensive stimulus trains on muscle fiber excitability and a protocol for intermittent repetitive stimulation suitable for fatigue studies. Recently, normative data based on a large sample of healthy subjects with a broad age-range was published to facilitate the future use of muscle excitability testing (Exl et al., 2025).

**Fig. 14:**
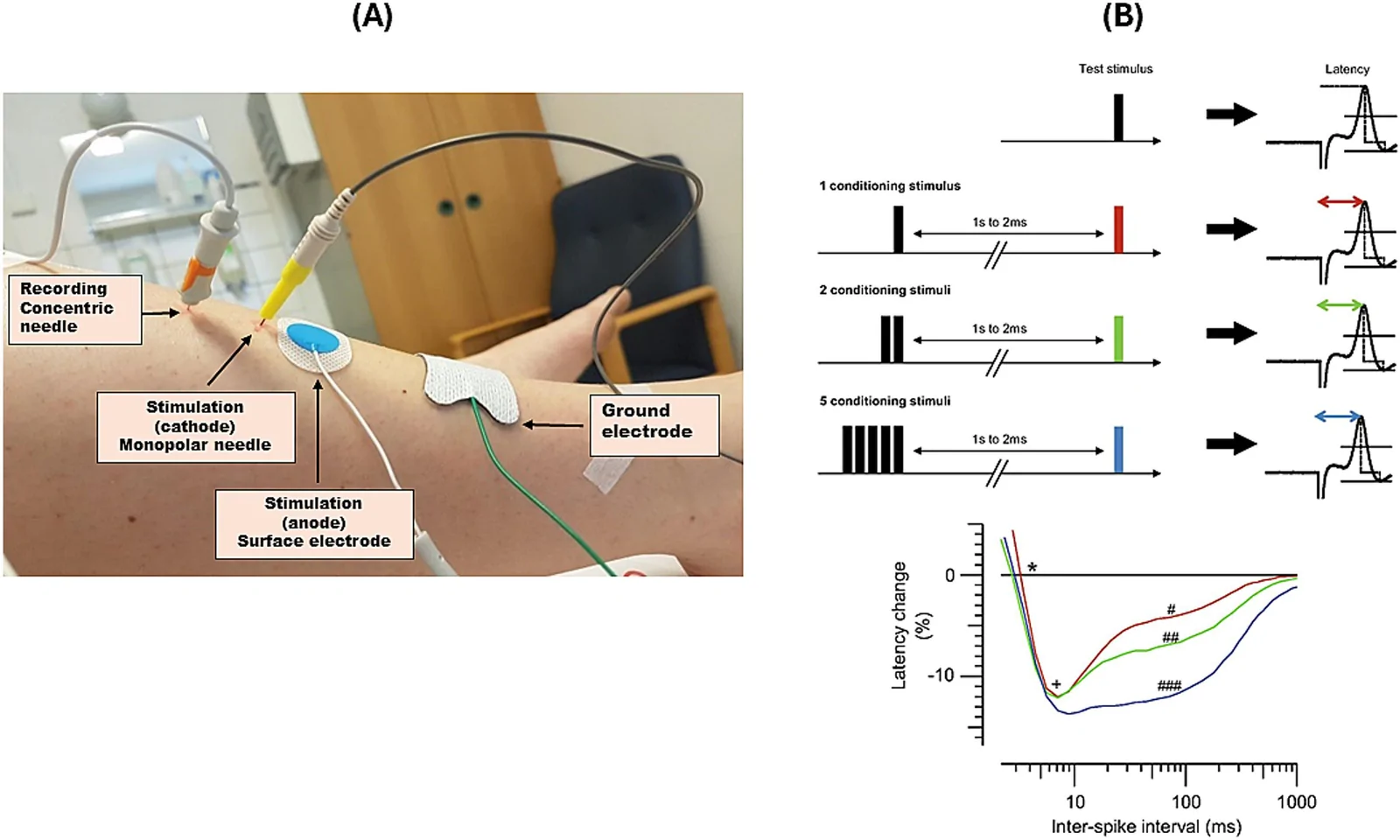
Illustration of muscle excitability measurements. A) Electrode arrangement for muscle excitability measurements. A monopolar needle serving as cathode is inserted perpendicular into the distal third of the tibialis anterior muscle. A surface electrode acts as anode and is placed further distally. Recordings from a concentric EMG needle 20 mm proximal to the stimulating needle. B) (right side, upper part) Multi-fiber muscle velocity recovery cycles (MVRC). Measures how preceding conditioning stimuli change, depending on the interstimulus interval, the velocity and hence the latency of a second muscle action potential elicited by the test stimulus. These changes provide an indirect indication of the after potential following the muscle action potential. This and the recovery cycle are dependent on membrane potential. (B) (right side, bottom part) Recovery cycle with percentage changes in latency of the response to the test stimulus due to 1 (red line), 2 (green) and 5 (blue) conditioning stimuli, plotted as a function of interstimulus interval. * muscle relative refractory period, + early supernormality, # late supernormality with one conditioning stimulus, ## late supernormality with 2 conditioning stimuli, ### late supernormality with 5 conditioning stimuli. Modified with permission from Rodriguez et al., (Rodriguez et al., 2021b). (For interpretation of the references to colour in this figure legend, the reader is referred to the web version of this article.)

### 10.3 Utility of muscle excitability testing

Since 2009, an increasing number of studies in healthy subjects and patients with neuromuscular diseases have been published by various research groups (for an overview please see (Tankisi et al., 2024a)). In summary, muscle excitability testing has the potential:

(i) to record disease-related changes in muscle membrane properties (Lee et al., 2019; Rodriguez et al., 2022; Tan et al., 2016; Tan et al., 2012; Tan et al., 2014; Tan et al., 2018; Z'Graggen et al., 2010), e.g., muscle channelopathies, critical illness myopathy and uremic myopathy.
(ii) to monitor disease evolution (Ackermann et al., 2014; Rodriguez et al., 2022),
(iii) to capture change of muscle membrane properties with a high temporal resolution in healthy subjects and in those with disease (Bostock et al., 2012; Hochstrasser et al., 2021; Larsen et al., 2021; Rodriguez et al., 2021a; Z'Graggen et al., 2010; Z'Graggen and Bostock, 2009; Z'Graggen et al., 2016; Zeppelin et al., 2023), e.g. during induced limb ischemia in healthy subjects or changes of serum potassium before and after hemodialyis.

Muscle excitability testing may be used in the future:

(i) to characterize diseases of striated muscle, to facilitate differential diagnosis and to simplify the diagnostic process.
(ii) to monitor disease progression and/or the effects of treatment.

Recently published results showed that it may enable early and confirmatory diagnosis of critical illness myopathy (CIM) (Rodriguez et al., 2022). Measured against the current diagnostic criteria, which require clinical and electrophysiological evaluation and muscle biopsy, muscle excitability testing had a diagnostic precision of 90% (AUC 0.908; sensitivity 1.000; specificity 0.714) at day 10 after intensive care unit admission for the confirmatory diagnosis and, importantly, could predict on day 2 after admission the development of CIM with a diagnostic precision of 82% (AUC 0.820; sensitivity 0.750; specificity 0.923). Muscle excitability testing may be used in future for confirmation of CIM and replace or complement the current diagnostic criteria, speeding up diagnosis and avoiding muscle biopsy. Perhaps even more importantly, muscle excitability testing could help select patients at risk of developing CIM for treatment trials. Muscle excitability testing is also of value in other myopathies, especially in muscle channelopathies (Tan et al., 2016; Tan et al., 2012; Tan et al., 2014; Tan et al., 2018).

### 10.4 Current limitations

Despite these promising results, muscle excitability testing is still primarily used in research. To facilitate clinical use the technique needs to be integrated into conventional EMG machines. Although no special hardware is required, none of the commercial EMG manufacturers have yet made this available and it still requires special equipment consisting of stand-alone stimulator, amplifier, data acquisition interface and a computer running the above-mentioned software. In addition, clinicians have not yet become aware of the technique, so it is only known within the scientific community despite its easy applicability and potential.

### 10.5 Conclusion

Muscle excitability testing is a new technique with great potential in the diagnosis and prognosis of diseases of striated muscle and for monitoring disease course through information on the properties of muscle fiber membranes, such as the membrane potential and ion channels. It is easy to perform at the bedside and fast. It has the potential to become part of clinical routine, especially testing is available on conventional EMG machines or an integrated stand-alone system.

## 11 Neuromuscular ultrasound

### 11.1 Introduction

For decades, physical examination, electrodiagnostic testing and tissue biopsy were the basis for diagnosing neuromuscular disorders. In the early 2000's, however, clinicians began to explore the use of ultrasound to assess the anatomical response of nerve and muscle to disease as well. Performed at the point-of-care and well-tolerated, ultrasound is now used routinely in the diagnosis of a number of conditions including entrapment neuropathies, CIDP, motor neuron disease, and varying muscle disorders. Further application of neuromuscular ultrasound has been limited by several factors including lack of standardization of imaging protocols and lack of a contrast agent, but these will be mitigated in the future.

Currently, clinicians rely upon B (brightness)-mode ultrasound to image nerve and muscle. These images provide structural information that can reveal disruption of normal tissue architecture due to oedema, compression from external structures, tumours and trauma. Reference values for peripheral nerve size have been established, helpful since many conditions result in enlargement (e.g. carpal tunnel syndrome, inflammatory neuropathies) or reduction (e.g. motor neuron disease, chronic sensory neuronopathies) in cross-sectional nerve area. Muscle is more variable and studies currently focus on identifying abnormalities in the brightness (or echogenicity) to identify areas of fibrofatty replacement of normal muscle tissue, which may be seen in myositis and dystrophies. However, attempts to quantify the brightness or echogenicity of nerve and muscle have been limited by differences in ultrasound systems and between operators' imaging. Additionally, lack of a contrast agent has prevented formal analysis of intraneural and intramuscular blood flow to aid in characterization of pathology.

### 11.2 Elastography

Ultrasound elastography is a non-invasive method of assessing tissue stiffness, often described as acoustic palpation. First developed in the 1990's, it is used currently in the diagnosis of hepatic disease and breast cancer (Cosgrove et al., 2013; Dietrich et al., 2017; Wee and Simon, 2019). Over the last two decades, there has been increasing interest in its use in neuromuscular disease. Peripheral nerve and muscle biopsies in various disorders show altered composition of healthy tissue, often revealing inflammatory changes, loss of normal architecture, fibrosis and in muscle, fatty infiltrates. These would be expected to affect tissue stiffness, creating a quantitative means of identifying pathology and possibly monitoring response to intervention. Elastography imaging allows both relative comparison between tissues and quantitative measures depending upon the elastography technique used.

All ultrasound elastography methods share the common mechanism of external force, either mechanical or acoustic, applied to body tissues. Strain elastography involves introduction of mechanical force by increasing the degree of pressure applied to the ultrasound transducer. It may also depend upon tissue displacement from cardiovascular or respiration pulsation, known as “ambient strain elastography” (Wee and Simon, 2019). In acoustic radiation force imaging, a pulse of ultrasound energy is directed into a region of interest (ROI) within body tissues and then the resultant tissue displacement is recorded. For all these methods, tissue displacement may be measured as a distance (often sub-millimeter) or a strain ratio. These methods all assume that the body tissues being imaged are homogenous and without boundaries, which is untrue in peripheral nerve and muscle.

Shear wave elastography imaging (SWEI) is the most promising method of assessing peripheral nerve and muscle. In SWEI, acoustic radiation force imaging is used to induce tissue excitation, but instead of measuring tissue displacement, the shear waves generated by this external force are tracked and measured. The shear wave velocity is measured in meters per second (m/s) or the results recorded as a shear modulus in kilopascals (kPa). Higher values represent increased tissue stiffness. SWEI is considered the most quantitative and objective form of elastography currently available (Neto et al., 2024).

Most work on SWEI in peripheral nerve disease has involved compression neuropathies, (CTS and ulnar neuropathy) and diabetic polyneuropathy (DPN). These studies demonstrate increased nerve stiffness in patients when compared with asymptomatic, healthy control subjects. Wu et al. (Wu et al., 2022) compared median nerve cross-sectional area (CSA) and SWEI measures at the distal carpal tunnel, carpal tunnel inlet, and forearm levels in 25 healthy volunteers and 54 patients with electrodiagnosis confirmed CTS. The patient group was imaged pre-operatively and one week post-operatively. At the forearm, there were no differences between the control and patient groups. However, the wrist sites revealed markedly increased nerve CSA and stiffness, most prominently at the carpal tunnel inlet preoperatively, with reduction in these values seen at 1-week post carpal tunnel release. These findings are typical of the work on compression neuropathies.

Since DPN is so common, there is much interest in quantitative methods for its early detection and for monitoring progression. Dong et al. published a meta-analysis of six studies that investigated the role of SWEI in DPN. These studies included a total of 198 patients with DPN, 168 patients with diabetes mellitus but no detectable DPN, and 154 healthy controls. Tibial nerve stiffness was measured at 4 cm proximal to the medial malleolus in all studies. The same ultrasound system was used in 5 of the 6 studies. Increased tibial nerve stiffness was demonstrated with an overall sensitivity of 75% and specificity of 86% for detecting DPN in those with the diagnosis. It may also have utility in diagnosing DPN prior to onset of clinical signs and symptoms. Wang et al. performed bilateral tibial nerve imaging in 41 patients with EDX-confirmed DPN, 42 patients with diabetes and no neuropathy (NDPN) and 21 healthy controls. Although there was no difference in nerve size between the groups (CSA), increased tibial nerve stiffness was present in the NDPN group compared to healthy controls. Peripheral nerve SWEI could be a valuable tool in the detection of preclinical DPN.

Although these studies are encouraging, we remain cautious about the quantification of SWEI data. The cut-off values for differentiating normal versus abnormal nerve varied considerably between the studies (2.6–4.1 m/s; 32.7–71.3 kPa), even though the same site was imaged in all studies and all but one study utilised the same ultrasound system (Dong et al., 2022). Additionally, this wide range of normative values reported for peripheral nerve SWEI is not isolated to studies on DPN. For example, other publications report marked differences in normative values for the ulnar (Bedewi et al., 2021; Paluch et al., 2018) and median nerves (Kantarci et al., 2014; Park et al., 2021). While some variability may be explained by differences in SWEI system software, transducer frequency, and settings, other factors must be considered. Placement of the transducer with respect to nerve orientation, proximity to bone (Bortolotto et al., 2017), and body mass index (Shang et al., 2023) all affect SWEI measurements. Undoubtedly, some of the issues are related to the SWEI's presumption that imaged tissues are homogeneous and without boundaries, while peripheral nerves are not.

Muscle SWEI faces many of the same challenges as peripheral nerve imaging, but the larger ROI is an advantage. For muscle SWEI, factors requiring standardization include limb positioning, the degree of muscle contraction, and the amount of pressure applied from transducer positioning (Creze et al., 2018; Kleiser et al., 2024). Age, body mass index and the degree of physical activity in the 24 h leading up to imaging may also affect results (Creze et al., 2018; Paramalingam et al., 2021). The angle of insonation (i.e. transducer orientation) can also alter the measured tissue stiffness by as much as a factor of four, a limitation that is being addressed by development of 3-D SWE techniques (Paley et al., 2023) (Fig. 15). Despite these limitations, muscle SWEI remains promising.

**Fig. 15:**
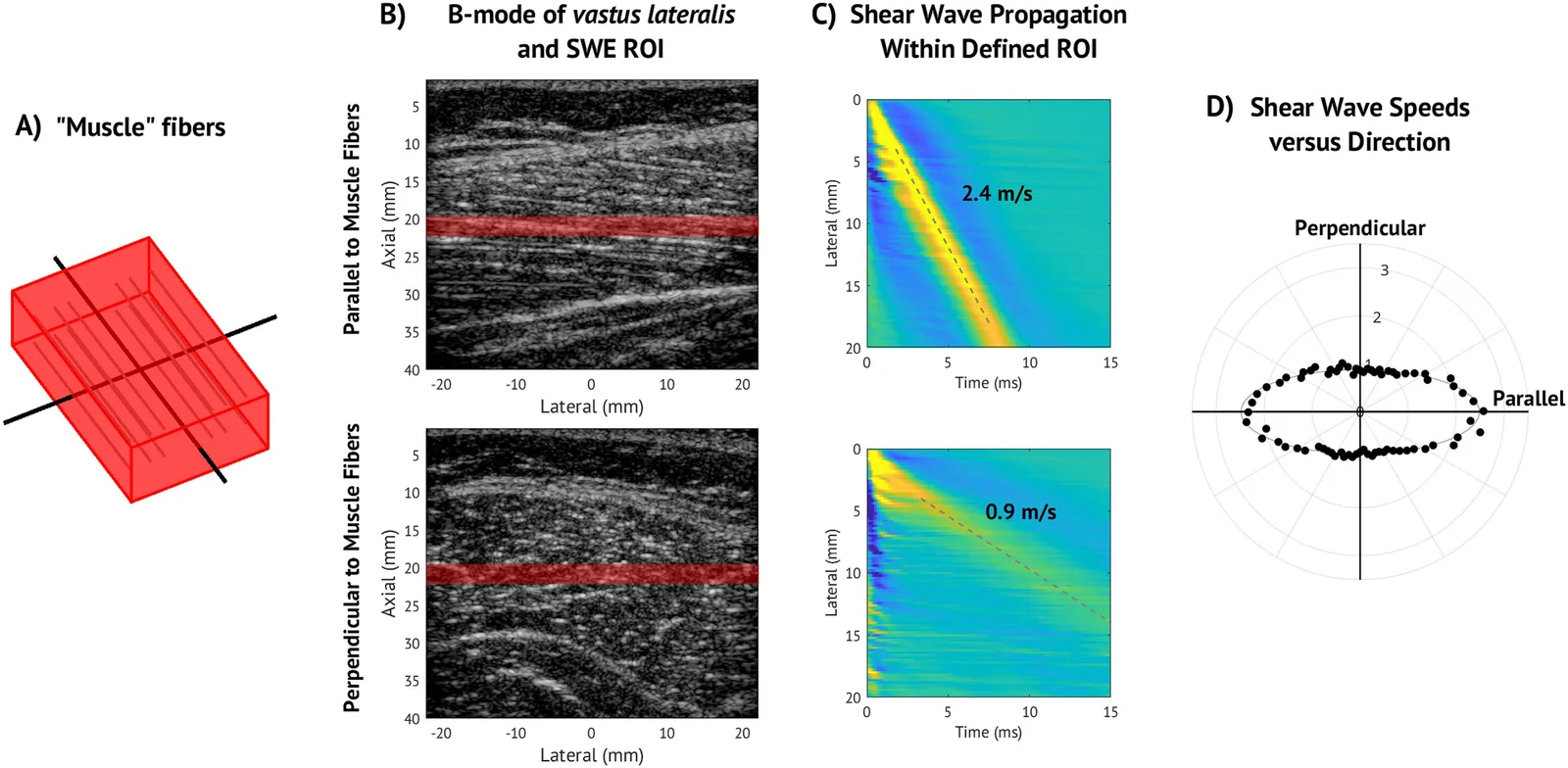
3D-shear wave elastography (3D SWE) techniques to characterize the direction-dependent mechanical properties of skeletal muscle. a) pictoral representation of skeletal muscle, displaying directions parallel and perpendicular to the muscle fibers. b) B-mode images of in vivo *vastus lateralis* and regions-of-interest (ROI) within the muscle body to conduct SWE measurements. c) shear wave propagation extracted from ROI, to quantify speed of shear wave through lateral position and time (m/s). d) Shear wave speeds plotted as a function of direction, showing large dependence of direction relative to the muscle fibers on SWE measurements. This 3D SWE technique is not available commercially, but is currently used in research to highlight the dependence of direction on musculoskeletal SWE measurements.

Published research on muscle SWEI primarily centers upon investigations of Duchenne muscular dystrophy (DMD) and inflammatory myopathies. In DMD, an increasing number of available treatments creates a need for point-of-care, non-invasive tests to monitor disease. Given the fibrofatty replacement of healthy skeletal muscle in progressive DMD, measures of tissue stiffness via SWEI seem well-suited for this purpose. Lin et al. (Lin et al., 2021) assessed lower limb muscle stiffness in 39 patients with DMD and 36 healthy controls, aged 3–20 years. The rectus femoris, tibialis anterior and medial gastrocnemius were imaged. In the early ambulatory stage, patients with DMD displayed increased muscle stiffness. However, DMD patients in the later, non-ambulatory stages displayed reduced stiffness in the tibialis anterior and medial gastrocnemius, while rectus femoris stiffness increased. Similar patterns have been noted in upper limb muscles (Lin et al., 2023). These early findings seem to indicate that while SWEI might be useful in monitoring longitudinal progression of DMD, the effects on individual muscles appears to vary.

Kolb et al. (Kolb et al., 2021) examined 21 patients with confirmed inflammatory myopathies and 24 matched, healthy controls. Both groups underwent SWEI, while the patient group also had muscle MRI. SWEI measures were elevated in the patient group, indicative of increased muscle stiffness. Affected muscles also showed increased heterogeneity of stiffness compared with healthy muscle. Areas of abnormality on SWEI correlated with abnormalities seen on muscle MRI. However, not all studies of inflammatory myopathy have shown increased muscle stiffness, with some showing it reduced (Alfuraih et al., 2019; Li et al., 2023). These differences may be related to the stage of disease with early, active inflammation more likely to be associated with increased muscle stiffness, but additional research is needed.

In summary, SWEI imaging is a promising technique for the diagnosis of peripheral nerve and skeletal muscle disease. It may also be useful in longitudinal monitoring of these disorders, though published works are awaited.

### 11.3 Optoacoustic Imaging

Optoacoustic imaging (OAI), sometimes known as photoacoustic imaging, is a novel technology that holds promise in peripheral nerve and muscular disease. OAI uses a non-ionizing laser to introduce energy into body tissues directed into a ROI where thermoelastic expansion occurs, generating sound waves. This acoustic energy then travels back to the OAI system, where light and sound are integrated to provide an image and information about the imaged tissue through spectral analysis (Regensburger et al., 2020a). This improves the spatial resolution of imaging and can provide information about tissue composition.

Jüstel et al. (Justel et al., 2023) performed OAI on the median, ulnar and radial nerves in 12 healthy volunteers. Vasa nervorum were visible on imaging and collagen, lipid and water content were visualized within the nerve. While no subjects with nerve disease were included in the study, differences in these components potentially may be used to diagnose peripheral nerve disorders, assess the degree of inflammation where relevant, and follow response to treatments. For example, detection of early changes in blood flow might be relevant in the diagnosis of early DPN.

OAI also has likely applications for muscular disorders with studies on DMD, Pompe disease and spinal muscular atrophy (SMA) already published. Proof of concept was established by Regensburger et al. (Regensburger et al., 2019) in DMD. After first validating measurements using a form of OAI known as multispectral optoacoustic tomography (MSOT) in a porcine DMD model, 10 patients with DMD and 10 matched healthy controls were imaged. In this study, collagen's signal was used as a marker of muscle fibrosis and 8 muscles were imaged per participant. MSOT detected fibrosis in 16.5% of images in children with DMD at 1 year of age as compared to only 3% of age matched controls. Additionally, clinically significant correlations were seen between MSOT-measured collagen content and clinical parameters, including age, strength, and the 6-min walk test.

Similar success has been seen using MSOT in imaging late onset Pompe disease (LOPD). Tan et al. (Tan et al., 2024)imaged the biceps brachii in 10 patients with LOPD and 10 healthy subjects, comparing the results with subjective gray-scale analysis of ultrasound and muscle MRI. Although the specific signal of glycogen could not be determined, the investigators found it was linked with the signal for water and used the latter as a surrogate marker. Muscle MRI fat fraction measures could not differentiate between healthy subjects and those with LOPD. Sonographers were 67% sensitive in detecting LOPD, comparable with MSOT findings. However, the study sample size was too small for any statistical significance to be determined.

Nedoschill et al. (Nedoschill et al., 2024) investigated MSOT for monitoring SMA patients' response to treatment. Again, this was a small study of 10 patients and 10 healthy, age-matched controls with both groups having a mean age of approximately 9 years. The investigators analyzed the overall signal intensity of imaged muscle and found the signal intensity to be reduced in SMA, most prominently in the proximal leg and increased following treatment. Strong correlations with clinical measures were noted in the patient population. This study provides early evidence that MSOT may be useful for monitoring response to treatment.

In summary, OAI provides novel information on nerve and muscle composition and may serve as an in vivo biomarker for diagnosing neuromuscular disease and monitoring response to treatment interventions.

### 11.4 Ultra-high frequency ultrasound

To date, most ultrasound studies of neuromuscular disorders have used high frequency probes (5 to 18 MHz linear transducers), which have limitations in image resolution. In recent years, ultra–high-frequency (UHF) ultrasound imaging systems that utilise 30- to 70-MHz transducer probes have been adopted in clinical studies, providing more detailed views of not only the nerve but its fascicles (Katzberg et al., 2017; Meiling et al., 2024b; Meiling et al., 2024c; Puma et al., 2019; Regensburger et al., 2020b). However, use of these probes is limited to more superficial structures due to poor depth penetration.

UHF ultrasound has been applied to study the median, ulnar, radial, fibular and sural nerves (Cartwright et al., 2017; Meiling et al., 2024a; Meiling et al., 2024c; Puma et al., 2024). These studies report reference values for fascicle counts and density in small cohorts of asymptomatic adults. Regarding neuromuscular disorders, UHF ultrasound demonstrates promise in immune-mediated neuropathies. In a study comparing HF vs UHF ultrasound probes for the evaluation of median and ulnar nerves in 11 CIDP patients, the authors found UHF ultrasound statistically superior in assessing nerve fascicle size and morphology, as well as in evaluating nerve vascularization (Puma et al., 2019). The same group later expanded the use of UHF ultrasound to differentiate between CIDP (12 patients), distal CIDP (6 patients) and anti-MAG neuropathy (10 patients). UHF ultrasound was reliable in differentiating these neuropathies from controls based on nerve and fascicular CSAs (Puma et al., 2024). Particularly impressive was the finding that fascicle ratio (fascicle CSA/nerve CSA) could differentiate distal CIDP from the other two neuropathies and that fascicle CSA was larger in CIDP and its variant than in anti-MAG neuropathy. In another study, Puma et al. imaged the sural nerve, comparing UHF ultrasound findings to histopathologic findings in 10 patients with severe, progressive polyneuropathies (Puma et al., 2021). They found UHF ultrasound had a high sensitivity but low specificity in identifying nerve inflammation.

UHF ultrasound may also be helpful in the assessment of motor neuron disease. A preliminary study of three children with SMA suggested that changes in median nerve fascicle number and density may correlate with disease severity (Regensburger et al., 2020b). In ALS, Meiling et al. imaged the median nerves of 15 patients using the 48-MHz linear array transducer, comparing these to fascicle counts previously reported in healthy controls (Cartwright et al., 2017; Meiling et al., 2024b). However, the authors failed to demonstrate a significant difference in fascicles between the groups.

The true potential of UHF ultrasound remains to be seen as, currently, studies have been limited to cross-sectional studies of small cohorts.

### 11.5 Artificial intelligence and machine learning

AI is making major impacts in medical imaging, including neuromuscular ultrasound. Specifically, machine learning (ML) techniques have improved the image quality and image acquisition speed, facilitating the diagnostic process. In ML, systems are trained by using large volumes of data and leveraging various types of algorithms. The three basic methods of ML are (i) supervised, where predictions are based on learned mapping, (ii) unsupervised, which analyses unlabelled data assuming certain properties, and (iii) reinforcement, where the information available in the training data is intermediate between supervised and unsupervised learning (Jordan and Mitchell, 2015).

In entrapment neuropathies, AI may improve automated detection of the nerve, as well as diagnostic accuracy in CTS. Ardakani et al. studied the performance of SWEI, morphometry, and AI techniques to accurately predict median nerve entrapment (Ardakani et al., 2020). Two ML techniques, i.e. the traditional support vector machine, and the deep learning (DL) convolutional neural network (CNN), were employed to study the morphology and elastic parameters of the median nerve in 200 wrists (100 CTS and 100 control). They found the CNN to be superior (AUC of 0.980) to support vector machine (AUC of 0.943) in identifying CTS.

DL-CNN has also been utilised to automate median nerve measurements and has high accuracy (90–95%) for calculating the CSA, thereby facilitating the diagnosis of CTS (Cosmo et al., 2021; Horng et al., 2020; Smerilli et al., 2022). Shinohara et al. also utilised DL to study CTS. However, rather than the CSA, they studied the echo pattern of the median nerve and surrounding tissue, finding an accuracy of 0.96 for detecting CTS, higher than reported accuracies with nerve CSA (Shinohara et al., 2022b). A similar study was performed in cubital tunnel syndrome using images from 30 patients and 30 controls. The authors found an accuracy of 0.90 in predicting cubital tunnel syndrome (Shinohara et al., 2022a).

These concepts can also be applied to muscular disorders. Using an algorithm based upon multiple DL architectures, Marzola et al. demonstrated that the CSA of certain transverse muscle ultrasound images can be accurately segmented (Marzola et al., 2021). The automated segmentation was then used to extract the mean grayscale values from a dataset that included 3917 images of biceps brachii, tibialis anterior and gastrocnemius medialis acquired on 1283 subjects. Both healthy and abnormal muscles were included, and the performance of the DL correlated highly with manually obtained values.

A similar study utilised DL-CNN together with vision transformers to segment muscle CSA in a different cohort. The authors studied 2005 transverse ultrasound images from four muscles (tibialis anterior, rectus femoris, gastrocnemius and biceps brachii/brachialis) recorded from 210 subjects of different ages, pathological conditions, and sexes (Katakis et al., 2023). They found minimal discrepancies between automation and manual measurements, further supporting the potential of DL in muscle ultrasound.

ML can also aid in the automated classification of muscle diseases (Burlina et al., 2017; Nodera et al., 2019). Burlina et al. tested ML and DL techniques in patients with inflammatory myositis and healthy controls (Burlina et al., 2017). The ML approach was semi-automated as the operator had to perform manual muscle delineation to generate images, whereas the DL-CNN approach was applied to the entire image (fully automated). DL-CNN was both more accurate (up to 87%) and more efficient. Nodera et al. used simple logistic, SMV and random forest classifiers to differentiate the texture analysis for 19 patients with myotonic dystrophy and 32 patients with inflammatory myositis (Nodera et al., 2019). Although the average pixel values were similar in both groups, they found significant differences for pixel standard deviation values, entropy histogram, gray-level length matrix and gray-level non uniformity level resulting in a classification accuracy of up to 78.4%.

As ML becomes increasingly integrated into ultrasound systems, AI models trained on large datasets of normal and abnormal neuromuscular ultrasound images will play an important role in helping clinicians reach a more accurate diagnosis and predicting disease progression. One of the limitations in US has been in its generation of normal and abnormal results on CSA etc. ML on large data sets may eliminate this problem.

## 12 Conclusions

Neuromuscular ultrasound is evolving rapidly. Improved spatial resolution with ultra-high frequency transducers represents a transition toward real-time microscopy, while techniques like elastography and optoacoustic imaging may take the role of contrast agents and provide non-invasive information on the composition of nerve and muscle in vivo. Artificial intelligence may improve scanning techniques and extract new information from images to improve diagnosis. The combination of these techniques may not only complement but also transform neurophysiologic testing in neuromuscular disease.

## 13 Conclusions and future perspectives

As scientists and clinicians, we are far more comfortable discussing results and data than less certain future developments. Despite this, authors of the ten sections above consider novels aspects of established clinical tools and some newer techniques.

The most common shared theme is the increased role for AI and machine learning in analysis of neurophysiology data to improve diagnostic and prognostic accuracy, and to allow data sharing between departments more easily. One important question is how the large data sets upon which AI depends will be assembled and made available. In its position statement on AI, prepared by Husain et al., the IFCN makes a plea for international large-scale cooperation rather than to have departments or companies leading.

Another shared aspect is a blurring of physiology and anatomical analysis, what might be called ‘physiological imaging.’ EIT is a non-invasive medical imaging technique that creates a real-time, tomographic image of the body's internal electrical conductivity. Used thus far to assess lung function, it can now be used in peripheral nerve to show selective fiber fascicles and then to allow focal vagal nerve stimulation to reduce side effects. EIM may become useful in spinal muscular atrophy, dystrophy, and ALS. Its flexibility may allow use in clinical trials with repeat assessment and home testing.

A second ‘physiological imaging’ technique is the measurement of magnetic fields associated with nerve or muscle activity; MNG and MMG. The emergence of optically pumped magnetometers has enabled recordings of electrical stimulation of peripheral nerves in nerve entrapments and in plexus and cord problems. In surface recording from muscles, it can record more motor units, from deeper areas, than surface EMG.

In the section on microneurography we learn that the adequate stimuli and electrical responses of neurons may change after damage. It is also now possible to relate ion channels expressed on dorsal root ganglion cells to the electrical properties of single neurons, thereby correlating genetic and neurophysiological characteristics of nerve cells and leading to the suggestion of there being sixteen sensory afferent cell populations. Microneurography has led to huge advances in understanding of peripheral nerve function and in the mechanisms of cutaneous perception. In careful hands this seems set to continue.

Excitability testing has led to greater understanding of peripheral nerve and muscle channelopathies. More widespread adoption has been held up by availability of the required software and EMG machines. Nerve excitability testing may also have a future role in the assessment of drug target engagement, therapeutic efficacy and disease progression. Muscle excitability testing may be useful on critical illness myopathy as well as in muscle hyperexcitability and nerve/muscle channelopathies. Since the technique is relatively simple to perform and is non-invasive, once limitations in availability and accessibility have been overcome then greater adoption may follow.

Ultrasound is now used routinely in many departments in the diagnosis of entrapment neuropathies and in CIDP and motor neuron disease. In the final section, various types of ultrasound, and their uses in nerve and muscle disease were discussed. Being non-invasive and quick, these tests are suited to repeat testing to assess disease progression and response to treatment.

With the new non-invasive tests described, it is indeed anticipated that repeat testing may become more routine, either to assess progression of a disease or its response to treatment. Clinical medicine is a harsh Darwinian world of natural selection where tests stand or fall by a complex mix of factors including speed, cost, sensitivity, and reliability. But occasionally too, an idea or a technique needs support in the face of initial disinterest. Champions are required for novel methods, and the sections above show some of these.

It will be fascinating to see how the field of peripheral neurophysiology evolves over the next years and which techniques make the leap from research to clinic. What the current chapter shows is the richness and variety of these new techniques, and the inventiveness, persistence, and passion for science their advocates demonstrate.

## Author contributions

Hatice Tankisi coordinated the chapter and wrote 2, 5, and co-wrote Section 3 with Hugh Bostock; Lisa D. Hobson-Webb and Nortina Shahrizaila wrote Section 11; David Holder wrote Section 6; James Howells wrote Section 9; Hakån Olausson wrote Section 8; Seward Rutkove wrote Section 7; Werner Z'Graggen wrote Section 10: Josep Valls-Sole wrote Section 4, and Jonathan Cole coordinated the chapter, wrote 1, 12, and critically reviewed the Chapter and language edited the manuscript.

## Declaration of competing interest

Bostock and Howells receive from UCL shares of the royalties for sales of the Qtrac software. Rutkove has equity in and serves a consultant and scientific advisor to Myolex, Inc. and Haystack Diagnostics; he is also a member of Myolex's board of directors.
